# A Pan-Genome Guided Metabolic Network Reconstruction of Five *Propionibacterium* Species Reveals Extensive Metabolic Diversity

**DOI:** 10.3390/genes11101115

**Published:** 2020-09-23

**Authors:** Tim McCubbin, R. Axayacatl Gonzalez-Garcia, Robin W. Palfreyman, Chris Stowers, Lars K. Nielsen, Esteban Marcellin

**Affiliations:** 1 Australian Institute for Bioengineering and Nanotechnology, The University of Queensland, Brisbane, QLD 4072, Australia; t.mccubbin@uq.edu.au (T.M.); r.gonzalezgarcia@uq.edu.au (R.A.G.-G.); r.palfreyman@uq.edu.au (R.W.P.); lars.nielsen@uq.edu.au (L.K.N.); 2 Corteva Agriscience, Indianapolis, IN 46268, USA; ccstowers@dow.com

**Keywords:** *Propionibacterium*, *Acidipropionibacterium*, *Cutibacterium*, propionibacteria, pan-genome, genome-scale model, re-annotation, propionic acid, RAST, KBase

## Abstract

Propionibacteria have been studied extensively since the early 1930s due to their relevance to industry and importance as human pathogens. Still, their unique metabolism is far from fully understood. This is partly due to their signature high GC content, which has previously hampered the acquisition of quality sequence data, the accurate annotation of the available genomes, and the functional characterization of genes. The recent completion of the genome sequences for several species has led researchers to reassess the taxonomical classification of the genus *Propionibacterium*, which has been divided into several new genres. Such data also enable a comparative genomic approach to annotation and provide a new opportunity to revisit our understanding of their metabolism. Using pan-genome analysis combined with the reconstruction of the first high-quality *Propionibacterium* genome-scale metabolic model and a pan-metabolic model of current and former members of the genus *Propionibacterium,* we demonstrate that despite sharing unique metabolic traits, these organisms have an unexpected diversity in central carbon metabolism and a hidden layer of metabolic complexity. This combined approach gave us new insights into the evolution of *Propionibacterium* metabolism and led us to propose a novel, putative ferredoxin-linked energy conservation strategy. The pan-genomic approach highlighted key differences in *Propionibacterium* metabolism that reflect adaptation to their environment. Results were mathematically captured in genome-scale metabolic reconstructions that can be used to further explore metabolism using metabolic modeling techniques. Overall, the data provide a platform to explore *Propionibacterium* metabolism and a tool for the rational design of strains.

## 1. Introduction

Propionibacteria are characterized by an unusually efficient anaerobic energy metabolism. This is primarily due to the Wood–Werkman cycle, which is a pathway that facilitates the production of propionate while maximizing energy capture [1,2]. Traditionally, the genus was composed of two groups, the classical or dairy propionibacteria and the cutaneous. However, recent phylogenetic analyses on newly available sequence data have led to an improved resolution of the phylogenetic relationships within this genus and a proposed reclassification of the taxonomy of several species [3], although there has been some resistance to the widespread adoption of this proposal [4]. Traditionally, dairy propionibacteria referred to species such as *P. acidipropionici* and *P. freudenreichii,* which are found in dairy products and the rumen and are associated with the ability to utilize a wide variety of carbon sources. They are used in various biotechnology applications: in particular, *P. freudenreichii* is used as a starter culture in cheese manufacture, as a probiotic, for the production of vitamin B12, and for the production of propionic acid for food applications [5,6,7]. Some *P. acidipropionici* strains have the potential to produce propionic acid at high titers and yields nearing those required to replace the chemical production of industrial-grade propionic acid [8,9]. This industrially relevant sub-group of propionibacteria has been reclassified into two genera: *Propionibacterium* (including *P. freudenreichii,* which no longer distinguishes between subspecies *freudenreichii* and *shermanii*) and *Acidipropionibacterium* (including former *P. acidipropionici)* [3]. The cutaneous propionibacteria group represents a distinct phylogenetic clade in the *Propionibacterium* genus and have been reclassified as *Cutibacterium* [3]. These species are opportunistic pathogens [10,11] and play an important role in skin health, inhabiting different regions of the skin reflective of their metabolic specialisation. For example, *P. avidum* inhabits moist, nutrient-scarce regions rich in sweat glands such as the axilla where they are likely dependent on proteolytic activity to supply amino acids as a major carbon and energy source, whereas *P. acnes* inhabits hair follicles and appears to predominantly rely on sebum degradation via lipases [10]. The final member of the newly proposed genera to update the traditional *Propionibacterium* genus consists of *Pseudopropionibacterium* and includes *P. propionicum*, which causes actinomycosis [12]. As this work compares the metabolisms of members from the newly adapted *Propionibacterium*, *Acidipropionibacterium*, *Cutibacterium,* and *Pseudopropionibacterium* genera, out of simplicity, we will use the terms propionibacteria and *Propionibacterium* to collectively refer to all of these genera and will not use the new classification to avoid confusion.

The prevalence of propionibacteria in the human microbiome has importance in human health and their industrial significance dictates a need for a thorough understanding of their metabolism. While many strains have been sequenced, accurate annotations are required to fully understand and ultimately rationally engineer metabolism in propionibacteria. The development of genome-scale metabolic models could offer new opportunities for studying propionibacteria, including the design of drugs or drug targets which could, in principle, be specifically targeted toward species involved in a secondary infection or acne vulgaris by studying species-specific metabolic capabilities and interactions with the human host [13]. In addition, such models would facilitate metabolic engineering, such as to enhance yields for the industrial-scale production of propionate [8,9] using knockout and overexpression vectors developed for a number of species [14,15,16]. Finally, they also offer a high-resolution insight into the metabolic adaptations that have occurred to allow commensal and dairy propionibacteria to occupy such differing habitats, and to assess the variability in metabolic capabilities of these organisms that has been hinted at in previous studies [17,18].

There are several challenges to producing accurate annotations for propionibacteria. Firstly, the high GC content of actinobacteria such as propionibacteria presents unique challenges to annotation, including a higher error rate and biased read coverage in genome sequencing [19], although recent long read technologies are helping to overcome this hurdle and drive the generation of new sequences [20]. Moreover, gene calling algorithms are susceptible to over-predicting gene length or failing to call genes [21]. Thus, automated annotations may miss many genes and inaccurately reconstruct others. Secondly, there is no biochemically well-characterized model *Propionibacterium* with the majority of metabolic activities in these organisms being inferred through homology rather than confirmed experimentally. Therefore, annotation by homology may produce erroneous substrate predictions. For example, homologues in two strains may be mapped to different functions. Finally, the genus is metabolically diverse. For example, the second major fermentation product, acetate, is produced by various pathways in different species, as illustrated when an attempt to enhance propionate production in *P. acidipropionici* [22] failed due to the targeting of an acetate pathway present in *P. freudenreichii* [23] but absent in *P. acidipropionici* [24]. A good functional annotation must capture both the characteristic features of the genus such as the methylmalonyl-CoA carboxytransferase and the unique features of the specific strain. At present, semi-automated reconstruction processes and annotation pipelines such as Rapid Annotation using Subsytem Technology (RAST) [25,26,27] are unable to accomplish this task, which is emphasized by a recent release of *Propionibacterium* models [28] that lacked a complete Wood–Werkman cycle and failed to accurately predict the different acetate production pathways.

To address these challenges, we developed a pan-genome guided metabolic network reconstruction of *Propionibacterium*. We extend the definition of the pan-genome to a genus basis as per several previous studies [29,30] and introduce the relatively novel concept of a genus-level genome-scale metabolic reconstruction, which summarizes the core and pan-metabolic functionality of the propionibacteria genus in a stoichiometric matrix, and to date, it has been performed in just a few studies without significant manual curation of the draft-metabolic reconstructions [31,32]. The metabolic reconstructions for closed *Propionibacterium* species were driven by functional cues arising from the draft metabolic models or strain and enzyme-specific information from the literature, and by genomic-context cues, which leveraged information from the pan-genomic matrix, and the local gene-context of orthologues for individual strains. *P. acidipropionici* ATCC 4875 was selected for deep experimental characterization, including growth on chemically defined media, proteomics, transcriptomics analysis, and phenotype arrays to drive further refinement of its annotation, which were subsequently propagated. Then, core and pan-metabolic reconstructions were generated from the individual highly curated reconstructions and utilized to compare the metabolism of five different species. Our approach demonstrates that synergistic complementation of pan-genome and pan-GEM (genome-scale metabolic model) analyses can overcome limitations in bioinformatics tools. The analysis revealed a possible evolutionary mechanism for the Wood–Werkman cycle and suggests a putative ferredoxin-linked electron bifurcating mechanism.

## 2. Materials and Methods

### 2.1. Strains and Gene Annotation

Of the 130 *Propionibacterium* genomes reported in the National Center for Biotechnology Information (NCBI) database at the time of performing the analysis, 21/01/2015, we focused the analysis on the 16 closed genomes due to reduced performance of the pan-genome approach when incomplete genomes are included [33], but note that metabolic reconstructions are less sensitive (Supplementary file 1, S1). Complete genomes of *Propionibacterium* strains were downloaded from the NBCI FTP server as GenBank files and consisted of two strains of *P. acidipropionici* (now *Acidipropionibacterium*), *P. freudenreichii* CIRM-BIA1, *P. avidum* 44,067 (now *Cutibacterium*), *P. propionicum* F0230a (now *Pseudopropionibacterium*), and 11 *P. acnes* strains (now *Cutibcterium*) (Table 1). Since the generation of quality metabolic reconstructions and pan-genomic comparisons is underpinned by correct and consistent gene annotation, the performance of alternative gene calling algorithms was evaluated in *P. acidipropionici* 4875 (Supplementary file 1, S2) using several metrics. GLIMMER was selected as the gene calling algorithm of choice, primarily because it resulted in the fewest intergenic peaks in RNA sequencing data, which were assumed to generally represent a failed call of a gene, and all genomes were annotated consistently and consecutively in the Department of Energy Systems Biology Knowledgebase (KBASE) pipeline [34].

### 2.2. Pan-Genome Comparisons and Matrix Generation

**Figure 1:**
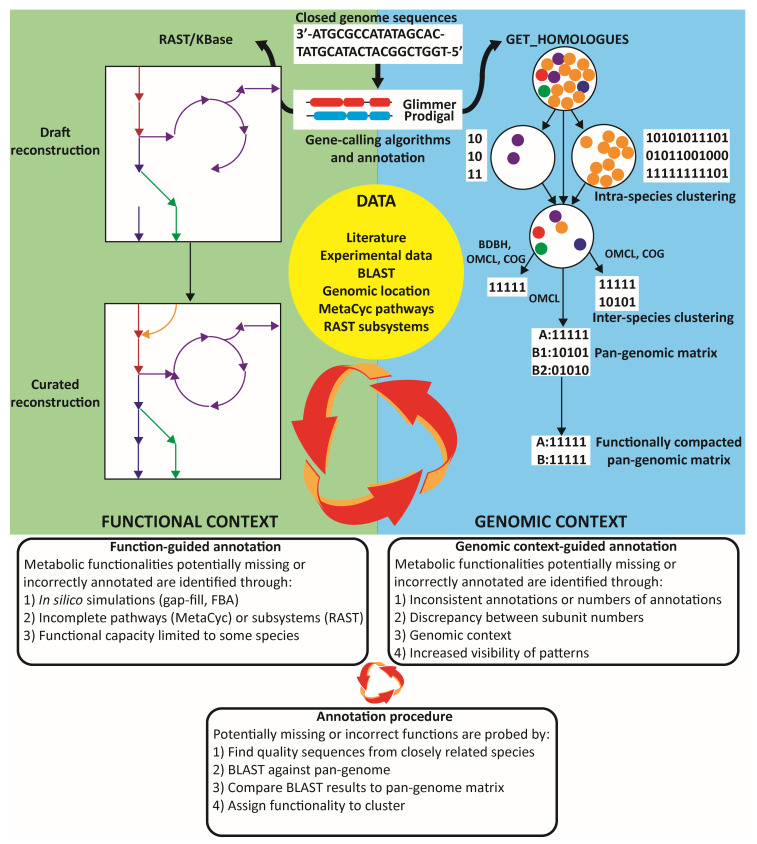
**Graphical depiction of the bioinformatics workflow.** Improved annotations of the genome, captured as a genome-scale metabolic reconstruction, are pursued using both functional approaches and genomic-context approaches that capitalize on information contained within the pan-genome.

A pan-genomic analysis underpinned the metabolic reconstruction effort by supplying extensive functional and sequence-based information (Figure 1). All clustering and pan-genomic analyses were performed on GenBank files generated from KBase using the Get_homologues package [33]. The analyses were performed on translated protein sequences with the OrthoMCL algorithm (OMCL) [46] with a granularity parameter of 1.5, minimum coverage of 75%, and maximum E-value of 10^−5^. Given there were multiple closed genomes for two species, the analysis was split into two stages. First, an intra-species analysis was performed, and representative strains for each species were selected for an inter-species analysis (Figure 1) to reduce the bias likely to arise from the intra-species variability exceeding the inter-species variability. The availability of additional experimental data made *P. acidipropionici* ATCC 4875 the ideal representative strain for *P. acidipropionici*, while *P. acnes* 6609 was chosen as the intra-species analysis suggested that it was the most functionally robust and therefore the most representative of the full metabolic functionality of *P. acnes*. Estimation of the core and pan-genome size was performed using the OMCL clusters with the random sampling implementation of the Tettelin [13] and Willenbrock [47] algorithms. Trees generated from Get_homologues were visualized using FigTree (available at http://tree.bio.ed.ac.uk/software/figtree/).

The orthologous clusters generated from the pan-genomic matrix were found to be poor representatives of the functional capacity of the genus. Evidently with a small set of strains of differing evolutionary distance, the algorithms will cluster true homologues in separate groups based on small sequence differences. Therefore, robust analyses, such as using the intersecting clusters of the OMCL, bidirectional best hit (BDBH), and COGtriangles algorithms [33], tended to discard key metabolic functionalities including from the Wood–Werkman cycle itself (Supplementary file 1, S3). Meanwhile, using just the OMCL algorithm still obscured approximately 96 core functionalities by differential clustering. Therefore, a functional annotation of the pan-genomic matrix was generated to complement the orthologous matrix in a two-step process (Supplementary file 2, S4) by combining entries with similar functional annotations, which was assisted in part with EC numbers where available and lists of synonymous enzymatic names primarily from the MetaCyc database [48]. Little effort was spent curating hypothetical and putative proteins, as these were expected to contribute negligibly to the functional understanding of *Propionibacterium* metabolism. Overall, the functional annotation collapsed the pan-genomic matrix by about two-thirds, while the number of core clusters remained roughly constant. Visualization of the orthologous and functional pan-genomic matrices was performed using Cluster 3.0 [49] and TreeView [50] (Supplementary file 1, S5).

### 2.3. Genome-Scale Metabolic Model and Pan-GEM Construction

Organisms selected for the pan-genomic analysis, as well as *P. acidipropionici* 55737, were further processed through the KBase pipeline to draft reconstructions (Figure 1). The metabolic models were initially gap-filled using chemically defined media formulations from experiments or the literature using the standard biomass template reaction or complete media formulations where these were not available. A chemically defined media was developed in house for *P. acidipropionici* that included several trace elements, glucose as a carbon source, nitrate as a nitrogen source, and phosphate as a source of phosphorous and the vitamins biotin, pantothenate, riboflavin, thiamin, and vitamin B12.

A consensus biomass equation was next constructed by datamining the available literature. Most investigations have focused on the unusual lipid and cell wall composition [51,52,53] of propionibacteria, but other studies on the macromolecular composition of *Propionibacterium* were absent. Therefore, we composed a consensus biomass equation for *Propionibacterium* that captures the predominant composition of the genus based on current knowledge, while acknowledging that there will be significant variability between different species; for example, the peptidoglycan structure is variable in the genus. DNA composition was determined from the genome sequence, and RNA composition was estimated based on the *P. acidipropionici* 4875 RNA sequencing data. Where unknown, the macromolecular composition and protein compositions were complemented with quantitative data from *Streptomyces coelicolor* [54]. After finalizing a biomass equation (Supplementary file 3, S6), previously added non-essential gap fills were removed.

The pan-GEM represents the entire annotated metabolic functionality of the individual reconstructions generated for each species. To create the pan-GEM, the individual metabolic reactions from each model are consolidated into a single supra-model, and additional columns are added to qualify the presence or absence of a reaction and the gene annotations within each representative species (Supplementary file 4, S7).

### 2.4. Pan-Genome Guided Genome-Scale Metabolic Reconstruction

The draft reconstructions developed were of low quality, containing numerous incorrect annotations and lacking key central carbon metabolic reactions including reactions of the Wood–Werkman cycle, highlighting the shortcomings of utilizing popular automated annotation pipelines for non-model organisms. A combined pan-genomic guided annotation approach was used to functionally correct the annotations by incorporating functional information contained within the models, pan-genomic information, and available data, and it is summarized in Figure 1. Briefly, the metabolic models gave functional cues to guide the annotation, such as gap fills, blocked reactions, differing metabolic capabilities between organisms, or testing catabolic capacity for experimentally verified nutrient sources, including phenotype array information. Metabolism was explored on a pathway basis using the pathways defined in MetaCyc [48] or subsystems from RAST to guide the search for potentially missing functionalities. Functional cues also come from the literature, which contains strain-specific information for certain genes and enzymatic capabilities. Another primary source of annotation cues comes from the genomic context of the orthologous and functional matrices. The genomic context benefits the annotation by enhancing resolution and allows the identification of inconsistent annotations, as well as potentially missed or incorrect annotations when they are present or absent in a small number of species, and it provides additional evidence for an annotation, such as if a function is flanked by genetic elements with related functions in some strains or highly conserved genomic regions. Analysis of the functional orthologous matrix allows the identification of reactions that were not captured in the draft models due to pipeline limitations and helps identify incorrect annotations such as inconsistent numbers of subunits of a particular complex. Then, potentially absent or incorrectly annotated genes were probed by identifying sequences from closely related strains and by using BLAST to compare these sequences against a local database. This database contained all modeled *Propionibacterium* protein sequences and genomes and could be used to query the propionibacteria pan-proteome or look for unannotated sequences in the pan-genome. Then, results are validated against orthologous gene clusters, and functionality is assigned on a cluster basis.

This manual curation approach was applied finitely and focused on pathways of the central carbon metabolism, particularly around the central phosphoenolpyruvate and pyruvate node and eventually expanded to include the respiratory metabolism and more specific metabolic traits of *Propionibacterium* including polyphosphate and trehalose metabolism. Particular attention was also given to amino acid metabolism, which can influence fermentation products [55]. By referencing the literature and the BRENDA database [56], known enzyme promiscuities that could fill functional gaps were identified, cofactor usage of reactions in the model was re-assessed and reaction reversibility was adjusted based on biochemical evidence and modeling results. For example, the irreversibility of the pyrophosphate generating reactions through the actions of intracellular pyrophosphatases was set in all models. For a brief guide of the manual curation of genome-scale metabolic models from the RAST framework, refer to S8 (Supplementary file 1).

### 2.5. Phenotype Array Data Generation and Integration

Phenotypic growth array data (Supplementary file 5, S9) for *P. acidipropionici* 4875 were generated and used to refine the model using an in-house gap-filling algorithm. The phenotype array and methodology was provided by Biolog, Inc. and was performed in accordance with their *Streptomyces* protocol with the modification of 2 µg/mL of known essential or growth-stimulating *Propionibacterium* vitamins: thiamine, biotin, pantothenic acid, riboflavin, and vitamin B12. All additives to the array were defined except the proprietary inoculating fluids. *P. acidipropionici* 4875 grew on 110 of the 189 carbon sources tested, of which 91 could be mapped to compounds in the ModelSEED database. Then, transporters for these 91 compounds were added to the *P. acidipropionici* 4875 model. Next, the complete database of ModelSEED reactions was used to test if each of these compounds could sustain growth, and where not possible, they were discarded from the analysis (14). Then, essential reactions required for growth on any of the conditions were identified and added to the analysis. Finally, the minimal number of reactions needed to sustain growth across all conditions was simultaneously computed using a mixed-integer linear programming formulation. A final round of manual curation was performed such that all reactions incorporated into the model based on this work were either supported by genes, enzyme promiscuity information, essential, or otherwise the only reasonable candidate, and results were propagated across other reconstructions where supported by genetic or biochemical evidence. The degradation pathways of phenylalanine and two related aromatic compounds completely lacked any genetic support for either of the currently known pathways for degradation and therefore were excluded.

### 2.6. Clustergram Generation

Comparison of the biosynthetic and catabolic potential of amino acids in propionibacteria was performed by generating clustergrams. Reactions associated with the catabolism or anabolism of each amino acid were grouped, and their presence or absence was scored in each reconstruction. Differences between strains were quantified using the Jaccardian similarity coefficient. Details of these pathways, brief functional assessments, and Jaccard score computation can be found in S10, Supplementary file 6. The Jaccardian similarity coefficient was utilized as a distance metric to generate the clustergrams in MATLAB.

### 2.7. Bioreactor Cultures and Omics Analysis

*Propionibacterium acidipropionici* ATCC 4875 was obtained from the American Type Culture Collection. Cells stored at −80 °C were used to inoculate serum bottles containing 50 mL PAM media (trypticase soy, 5 g/L; yeast extract, 10 g/L; K_2_HPO_4_, 0.05 g/L; MnSO_4_, 0.05 g/L with 40 g/L of the relevant carbon source). Cultures were incubated statically at 32 °C for 24 h and used to inoculate Applikon Ez-Control bioreactors containing PAM and 100 g/L of glucose, sucrose, or glycerol. All experiments were performed in duplicates. Reactors were maintained at 32 °C, pH 6.5 with 5 M NaOH, agitated at 200 rpm, and the head space was degassed with nitrogen to maintain anaerobic conditions throughout the fermentation. Regular samples were taken to measure optical density using a Biochrom Libra S12 spectrophotometer and for organic acid analysis. Cells were harvested for RNA and protein extraction during the mid-exponential phase. Total RNA and mRNA enrichment, sequence alignment, and differential expression analysis were performed as described in [57]. For proteomics, cell pellets were lysed and trypsin digestion was performed as previously described in [58] and quantified using sequential window acquisition of all theoretical mass spectra (SWATH) based quantification performed as described in [59], using a Shimadzu Prominence nanoLC-MS/MS connected to a Triple-ToF 5600 mass spectrometer (AB SCIEX, Framingham, MA, USA). The differential abundance of proteins with more than 2 peptides with a 95% confidence score and a false discovery rate less than 1% was assessed using the LIMMA package. Extracellular organic acids, carbohydrates, and alcohols were quantified by ion-exclusion chromatography as described in [60].

### 2.8. PFOR Knockout

**Figure 2:**
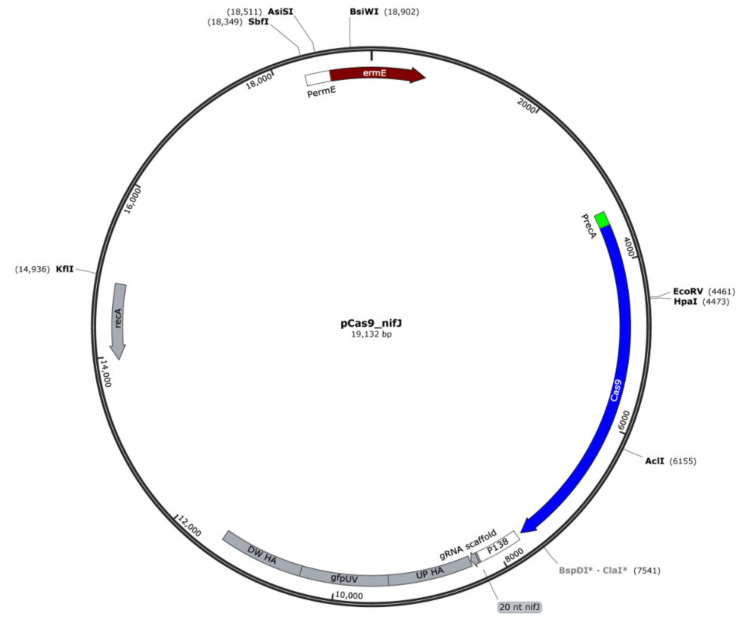
Plasmid map for pCas9_nifJ. Knockout is driven by recombination between downstream and upstream homologous arms (DW HA and UP HA, respectively) and the *nifJ1* gene in the chromosome, leading to disruption of the *nifJ1* with gfpUV.

The pyruvate:ferredoxin oxidoreductase (PFOR) encoded in *nifJ1* gene (PFREUD_RS00925, CDS.199 this study) was knocked out in *P. freudenreichii* using CRISPR/Cas9 by interruption of the gene with gfpUV. Briefly, we assembled plasmid pPAC_Cas9 using pRGO1 as the plasmid backbone and introducing the lacZ-MCS region, an erythromycin resistance gene (*ermE*), and a GC-optimized Cas9 under the control of promoter PermE. Then, Gibson assembly was used to create the knockout vector, pCas9_nifJ (Figure 2), by adding homologous arms designed to span 1 kb up and downstream of a PAM region identified in the *nifJ* gene, the gfpUV sequence, and gRNA under the control of the P130 promoter from propionibacteria. The homologous arms and gfpUV were PCR amplified from *P. freudenreichii* and the pBRPprp_gfpuv plasmid respectively. Primer sequences and the 20-nt region for the gRNA are shown in Table 2. All cloning was performed in *E. coli* DH5α (Bioline). PCR reactions were performed using Phusion Taq (NEB). Introduction of plasmid pCas9_nifJ into *P. freudenreichii* was performed by electroporation as previously described [61]. After 3 h recovery on PAM media with 40 g/L sucrose, cells were plated on NLB (sodium lactate, 10 g/L; trypticase soy, 10 g/L; yeast extract, 10 g/L; glucose, 10 g/L) agar and incubated in an anaerobic jar at 30 °C. After 10 days, colonies were identified and tested by colony PCR to confirm the successful knockout. Positive ∆nifJ colonies were then grown on NLB liquid media for 72 h.

## 3. Results and Discussion

### 3.1. Exploration of Core and Pan-Genome Size

**Figure 3:**
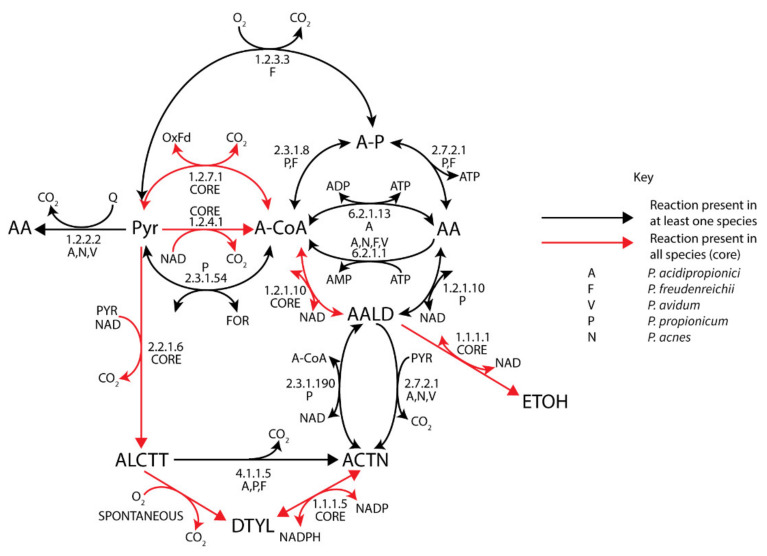
Acetate, acetoin, and ethanol metabolism of *Propionibacterium*. (Black) arrows represent reactions present in at least one species. (Red) arrows represent reaction in all species (core).

**Figure 4:**
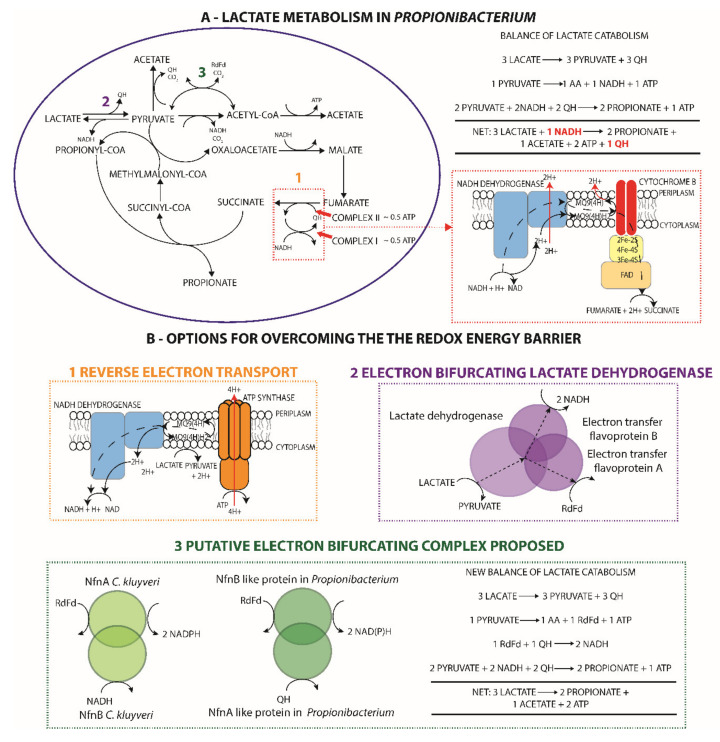
A novel ferredoxin-based energy conservation mechanism explains discrepancies between the literature and observed lactate metabolism in *Propionibacterium*. (**A**) Catabolic pathways for the conversion of lactate to propionate and acetate are illustrated and are most representative of the dairy propionibacteria, particularly *P. acidipropionci*, where the 2:1 ratio of propionate to acetate has been observed. The anaerobic electron transport chain is illustrated (bordered in red) and can be entered at complex II (cytochrome B) via quinols, presuming the translocation of two protons or half an ATP gain in energy, or complex I via NADH netting an additional two protons pumped. The balance over lactate catabolism illustrates that an overall redox imbalance exists where a quinol must be used to regenerate an NADH. (**B**) Potential strategies for overcoming this redox challenge are illustrated and are referenced to the appropriate location in metabolism in A. There was no genetic evidence for the first two mechanisms in propionibacteria; however, the last step is proposed based on similarities with flanking genes of the pyruvate ferredoxin oxidoreductase and the Nfn complex of *Clostridium kluyveri*, which could allow for the regeneration of NADH from a quinone.

While it is an integral feature of the reconstruction process developed here, the pan-genomic analysis also gives a preliminary estimate of the extent of diversity inherent in *Propionibacterium*. Unlike previous genus level pan-genomic analyses [29,30], only a single representative from each species was utilized to avoid the clustering bias likely to arise from a greater similarity between strains. The pan-genome of the five species studied was composed of 6852 clusters, 876 (13%) of which composed the core *Propionibacterium* genome and 4445 (65%) of which were strain-specific (Supplementary file 1, S5). Extrapolating from this analysis using the Tettelin [13] and Willenbrock [47] algorithms, we estimated that the core genome was between 792 and 906 clusters, while the pan-*Propionibacterium* genome was open, with an additional 553 new orthologous gene families identified per new species sequenced. Together, these analyses highlight significant variability between different propionibacteria species, reflecting the proposed taxonomic reclassification [3], while phylogenetic trees also demonstrate significant intra-species variability (S5, Figure 3 and Figure 4, Supplementary file 1).

### 3.2. Proteomics and Transcriptomics of P. acidipropionici

As part of the deep characterization of *P. acidipropionici* ATCC 4875 as a reference propionibacteria strain, proteomics and transcriptomics data were gathered on glucose, sucrose, and glycerol carbon sources. Each carbon source displayed key phenotypic differences; glycerol fermentation is essentially homofermentative for propionate, while the sugars produce substantially more acetate, succinate, pyruvate, and lactate by-products (Supplementary file 7, S11). Differences between glucose and sucrose fermentation were more subtle; the propionate-based yields of pyruvate and acetate halved and doubled, respectively. Differences in the phenotype between two conditions were compared with the differentially abundant transcripts and proteins in order to infer possible roles of these genes in broader metabolism and are discussed where relevant in the manuscript. Over all conditions, 3412 transcripts (≈99% of the genome) were detected with a reads per kilobase million (RPKM) > 1 and 1085 proteins were relatively quantified, covering 100% and 63% of the core orthologous clusters previously identified respectively, and 9% of genes (313) were found to be differentially expressed at the transcriptome or proteome level.

While outside the scope of this work, we note that a deeper analysis of the ‘omics data in this study may help guide further research into regulation and metabolic engineering efforts to improve propionate production. For example, the contrast between glycerol and sugars provides over-expression candidates to improve propionate production, several of which have already been experimentally validated [63,64], while the fumarate reductase and NADH dehydrogenase indicate that the electron transport chain may be a novel upregulation target. This same contrast may also be informative for studying nitrogen regulation in propionibacteria with nitrogen metabolism and nitrogen respiration extensively downregulated on glycerol (Supplementary file 7, S11). The correlation between the transcriptome and proteome can also identify over-expression targets unlikely to be under transcriptional control, with only 36.5% of proteins having a strong correlation to their transcript (R^2^ > 0.75) (Supplementary file 7, S11).

### 3.3. Model Modifications Summary

The pan-genomic matrix is insufficient to compare the metabolic functionalities of different propionibacteria due to factors such as convergent evolution, lateral gene transfer, and the splitting of true orthologues due to different evolutionary distances between organisms from different genera. A functional pivot of this matrix can surpass some of these issues, but it suffers from a lack of manual curation, particularly in the context of a metabolic network. Therefore, metabolic reconstructions of five *Propionibacterium* species were generated through a novel workflow that leverages information from the pan-genomic matrix and its functional pivot (as detailed in the methods section). This included two *P. acidipropionici* strains, which are the parents of high propionate producing genome-shuffled mutants [60,65] with industrial relevance and to give a sense of intra-species variation. Individual reconstructions were combined to create a genus-level reconstruction.

The scope of the manual curation of draft reconstructions derived through KBase using this workflow is captured in Table 3 and Table 4. On average, reconstructions were expanded in reactions and transporters by 30% (5% lacking gene assignment) and 120% (47% without gene assignment), respectively. The benefits of the pan-genomic approach are observed in Table 3 where the relative increase in information of the reconstructions of commensal species is equal to or greater than the more highly characterized dairy species. In the final reconstructions, the dairy strains had no essential nutrient requirements other than the two vitamins required by all *Propionibacterium*, biotin, and pantothenate (Table 5). Commensal strains had more complex requirements, requiring additional vitamins and several amino acids in the case of *P. acnes* and the nucleotide triphosphates UTP and CTP for *P. propionicum*. Reconstructions are available as an Excel file with corresponding models available as SBML files and original GenBank files in Supplementary file 4. The *P. freudenreichii* model was shown to be predictive for the design of genetic engineering strategies in a recent publication [61].

### 3.4. The Metabolism of Propionibacteria

The genus-level metabolic reconstruction consisted of 1223 metabolic reactions and 279 transporters, with the core reactome consisting of 761 metabolic reactions. Of the metabolic reactions, about 16% (124) occurred only in dairy species with 3% (23) occurring in all dairy propionibacteria analyzed. Conversely, 95 reactions (13%) were only present in cutaneous species with 16 occurring in all cutaneous species. Small differences were observed between the two *P. acidipropionici* strains, 20 metabolic reactions were present in 55737 and absent from 4875 while 16 occurred in 4875 only; however, 13 of these functionalities were added on the basis of phenotype array data and could not be assigned to any genes. The number of species-specific metabolic functionalities was variable; 73 reactions in *P. acidipropionici* (59 gene-associated), 45 in *P. propionicum*, 21 in *P. freudenreichii,* 15 in *P. avidum* and just 3 in *P. acnes*. The lower number of specific functionalities for *P. avidum* and *P. acnes* is hardly surprising given they have both been reclassified as *Cutibacterium* and are therefore, they are the only genus with two different species included in the analysis. Accounting for this, we found that *Cutibacterium* possessed 40 unique functionalities. To better understand how the metabolism of the 5 species analyzed is conserved and variable, the genus-level metabolic reconstruction was used to compare central carbon metabolic pathways and key metabolic nodes, namely redox metabolism, the pyruvate and phosphoenolpyruvate node that splits metabolism and regulates carbon flow, the characteristic trehalose and polyphosphate metabolism, and amino acid metabolism.

#### 3.4.1. Central Carbon Metabolism

Complete pathways for glycolysis, the Wood–Werkman cycle, and the pentose phosphate pathway were found in all genomes with the exception of *P. avidum*, which did not have a transaldolase (2.2.1.2). Three variations of the TCA cycle lacking a glyoxalate bypass were observed which differed in how 2-oxoglutarate was dissimilated to succinate, either with substrate-level phosphorylation via the succinyl-CoA ligase (6.2.1.5) (in *P. acidipropionici*, *P. avidum*, and *P. acnes*), or without via a multifunctional *menD* enzyme encoding the 2-oxoglutarate decarboxylase (4.1.1.71) and succinate semialdehyde dehydrogenase (1.2.1.16) identified in all studied propionibacteria or a complete GABA shunt confined to the dairy species. The dairy species also contained alternative central carbon metabolic pathways absent in commensal species studied. *P. freudenreichii* can catabolize glucose through the *Bifidobacterium* shunt, which favors acetate production and may explain the lower ratio of propionate produced relative to acetate observed in this strain [61,66]. *P. acidipropionici* possesses and transcribes a linear variation of the Wood–Werkman cycle that utilizes a sodium-pumping methylmalonyl-CoA decarboxylase with a similar structure to that of *Propionigenium modestum* [67] (mean ≈ 67 RPKM, Supplementary file 7, S11). This complex, in combination with the methylmalonyl-CoA mutase (5.4.99.2), epimerase (5.1.99.1), and the propionyl-CoA:succinate CoA-transferase (2.8.3.-), facilitates the generation of a membrane potential through the linear dissimilation of pyruvate to propionate via succinate and potentially pre-dated the more energetically efficient Wood–Werkman cycle. Indeed, the α subunit of the complex and 12S subunit of the unique methylmalonyl-CoA carboxyltransferase (2.1.3.1) of propionibacteria have high identity.

The ability of *Propionibacterium* to degrade different sugars is of interest for the renewable production of propionic acid. We briefly surveyed the capability of *Propionibacterium* to degrade the constituents of hemicellulose, which is a major component of plants, as well as sucrose. All strains could degrade mannose and were likely capable of catabolizing arabinose through the pentose phosphate pathway, although an arabinose proton symporter was only found in the dairy species. The capability to degrade xylose was only found in *P. acidipropionici* species. *P. acidipropionici* 4875 was found to contain a xylose isomerase and xylulokinase; however, a xylose isomerase could not be found in strain 55737. Given that we observed the growth of this strain on xylose as a sole carbon source (unpublished results), promiscuous activity of the rhamnulose isomerase is likely able to compensate for this. Sucrose-specific degradative capabiltiies were only observed in *P. acidipropionici* and *P. propionicum*, which both contained a 6-phosphosucrose fructohydrolase, allowing the catabolism of sucrose via the PTS system, as well as a sucrose-hydrolyzing levanase. Both mechanisms are likely to be active in *P. acidipropionici* given upregulation of the 6-phosphosucrose fructohydrolase, fructose PTS, and phosphofructokinase on sucrose (Supplementary file 7, S11). A less efficient catabolism of sucrose likely occurs in other strains, as observed in *P. freudenreichii* [68], due to the promiscuous hydrolysis activities of enzymes including amylosucrase and α-glucosidase. Indeed, α-glucosidase was also upregulated in *P. acidipropionici* grown on sucrose (Supplementary file 7, S11). Another feature of potential industrial relevance is the *pdu* operon, allowing the carboxysome-mediated degradation of 1,2-propanediol to propionate or glycerol to 3-hydroxypropionate [69] found in *P. freudenreichii*, which is in agreement with previous observations [23,70], as well as *P. propionicum*. With this general overview of central carbon metabolism, we now focus in on the key nodes.

#### 3.4.2. Acetate, Ethanol, and Acetoin Metabolism

Acetate metabolism is surprisingly variable between different propionibacteria (Figure 3), despite acetate being a major fermentation by-product and acetate-associated reactions acting promiscuously on propionate equivalents. In fact, promiscuous activity of the propionyl-CoA:succinate CoA-transferase from the Wood–Werkman cycle is the only common enzyme allowing acetate production in propionibacteria, suggesting that other pathways have been acquired through a method such as lateral gene transfer. All species could synthesize acetyl-CoA via the pyruvate flavodoxin oxidoreductase (PFOR) (1.2.7.1) or pyruvate dehydrogenase (1.2.4.1), while a pyruvate formate lyase (2.3.1.54) was only identified in *P. propionicum*. Further conversion to acetate with concomitant substrate-level phosphorylation was restricted to *P*. *freudenreichii* and *P*. *propionicum* via a phosphoacetyltransferase and acetate kinase (2.3.1.8), which was potentially inherited with the *pdu* operon, or the ADP-dependent acetyl-CoA synthetase (6.2.1.13) in *P. acidipropionici*, which is co-located with the sodium-pumping methylmalonyl-CoA decarboxylase. Propionibacteria lacking the *pdu* operon could directly produce acetate via a pyruvate:quinone oxidoreductase (1.2.2.2), which despite its energetic inefficiency is likely the major source of acetate in *P. acnes* and *P. avidum*, suggesting these strains are more reliant on aerobic respiration. This enzyme had a higher transcription in *P. acidipropionici* grown on glycerol where there is minimal acetate production, suggesting that the role of this enzyme is likely for redox balancing, whereas the energetically beneficial acetyl-CoA synthase is upregulated on sugars (Supplementary file 7, S11). Notably, *P. freudenreichii* contains an alternative pyruvate oxidase (1.2.3.3) that consumes oxygen and produces acetyl-phosphate, which likely serves as an energy yielding oxygen-tolerance mechanism. This is consistent with the observation of higher acetate production and propionate degradation when *P. freudenreichii* was exposed to oxygen [71].

A lack of ethanol and propanol production has been observed in *Propionibacterium* fermentations, even though all strains encoded alcohol dehydrogenases (1.1.1.1) and contained proteins that can give acetaldehyde dehydrogenase (1.2.1.10) functionality. This suggests that these enzymes either are repressed or have evolved tighter substrate specificity. Notably, *P. freudenreichii* and *P. propionicum* have acquired a propanol dehydrogenase with the *pdu* operon [72]. Only *P. propionicum* appears to contain a true acetaldehyde dehydrogenase and has the hallmarks of an ethanol-producing strain including the pyruvate-formate lyase; therefore, it would be an attractive candidate to screen for propanol production. Acetaldehyde is also a precursor for acetoin production associated with the formation of eyes in cheese. All strains could produce acetoin aerobically via diacetyl (1.1.1.5), but they utilized different paths anaerobically, either via acetolactate (2.2.1.6) or acetaldehyde (2.7.2.1) or both for *P. acidipropionici* (Figure 3). *P. propionicum* was the only species containing a degradation pathway for acetoin (2.3.1.190).

#### 3.4.3. Lactate and Ferredoxin Metabolism

Lactate is a key carbon source that is preferentially catabolized over glucose in some species [73], and its fermentative production plays an important role as a metabolic valve when the oxidation of NADH by the respiratory chain is limiting [74,75]. The highly endergonic production of lactate is facilitated by an NADH-dependent lactate dehydrogenase (1.1.1.27) found in all strains. Lactate oxidation is facilitated by either a menaquinone-dependent lactate dehydrogenase in *P. propionicum* or a core set of three proteins first characterized in *B. subtilis* [75] and *Shewanella* [76], which likely couples lactate oxidation to fumarate reduction via quinones or allows the direct reduction of cytochrome B, albeit weakly [77,78]. We observed upregulation of the aforementioned complex along with a lactate permease on sugars, where a lactate overflow was observed, relative to glycerol in *P. acidipropionici* 4875; this finding is in line with previous observations of transient lactate production before reconsumption during sugar fermentations in this strain [8].

Despite the extensive study of lactate fermentation by propionibacteria, no attention has been given to the energetic challenge of anaerobic lactate catabolism originally highlighted in 1973 [2]. Three lactate molecules are fermented to form two propionate and one acetate molecule [2,79] in accordance with the redox balance. However, this necessitates an energetically unfavorable reduction of NAD by a quinol; three lactate molecules are oxidized to pyruvate, generating three quinols, two pyruvate molecules are reduced to two propionate molecules requiring two NADH, and two quinols (in the absence of the NADH dehydrogenase), and the remaining pyruvate is oxidized to acetate with the generation of a quinol, NADH, or ferredoxin, as previously discussed (Figure 4A).

Given that propionibacteria lack an alternative complex I permitting reverse electron transport driven by ATP hydrolysis to reduce ferredoxin or NAD with a quinone [80,81] (Figure 4B), a ferredoxin-based energy conservation mechanism appears to be the most likely solution. This is further supported by proteomics and transcriptomics data for *P. acidipropionici* 4875, which surprisingly suggests that ferredoxin plays a major role in central carbon metabolism. Both datasets show significant upregulation of the PFOR on sugar, which appears to be the predominant source of acetyl-CoA given that expression is 2–3 times higher than the pyruvate dehydrogenase (Supplementary file 7, S11). Conversely, expression of the pyruvate dehydrogenase is four times higher than the PFOR on glycerol, illustrating that ferredoxin is associated with acetate production and not growth. Despite this, homologues to known complexes that could solve this energetic challenge could not be identified; namely, an electron confurcating lactate dehdyrogenase was identified in *Acetobacterium woodii* [82] (Figure 4B) or the FixABCX complex in *Azotobacter vinelandii*, which reduces ferredoxin and a quinone by oxidizing 2 NADH [83].

Although no homologous genes were identified for the FixABCX complex, we postulate that a similar complex exists in propionibacteria acting in the reverse direction. The flanking genes of the PFOR were identified as candidate genes for this activity based on evidence from bioinformatics analysis, ‘omics data, and genetic knockout. Our attention was drawn to these genes because they were co-transcribed with the PFOR (Supplementary file 1, S12) and were regulated identically at the protein and transcript level on different carbon sources (Supplementary file 7, S11). Although they were annotated as a glutamate synthase (NADPH) small chain/pyridine nucleotide-disulfide oxidoreductase and a dihydroorotate dehydrogenase in all genomes, we noted that the Nfn complex in *Clostridium kluyveri*, catalyzing the ferredoxin-mediated reduction of NADP+ in the presence of NADH [84], was originally assigned the same annotations. A domain analysis revealed that the glutamate synthase-like gene had a similar domain composition to *nfnB* consisting of two 4Fe-4S clusters and NADP+ and FAD-binding domains, but it also contained an additional ferredoxin-binding site, whereas the dihydroorotate dehydrogenase-like gene showed weak similarity to the *nfnA* of *C. kluyveri* and a higher similarity to type II dihydropyramidine dehydrogenases, which are directly associated with quinones. A CRISPR-mediated gene knockout in *P. freudenreichii* of the PFOR flanked by these putative genes severely crippled growth, despite other potential metabolic sources of ferredoxin and a duplicated PFOR. This suggested that this PFOR was the primary source of ferredoxin in the cell and ferredoxin plays an important role in maintaining a high growth rate. On this basis, we postulate that the two aforementioned genes and the PFOR form a complex with energy-conserving electron bifurcating properties, where ferredoxin and NAD(P) bind to the NfnB-like subunit and the NfnA-like subunit oxidizes quinols instead of ferredoxin. Further studies are warranted to confirm the existence of such a complex.

#### 3.4.4. Pyruvate Metabolism

Excluding the phosphotransferase system, three separate mechanisms were identified allowing the inter-conversion of pyruvate and phosphoenolpyruvate (PEP). All *Propionibacterium* contained the pyruvate kinase (2.7.1.40) and the gluconeogenic pyrophosphate-linked pyruvate phosphate di-kinase (2.7.9.1), which is a role supported by the latter being upregulated on glycerol in *P. acidipropionici* (Supplementary file 7, S11). *P. acidipropionici* and *P. propionicum* also contained the energetically more expensive PEP synthase (2.7.9.2), which plays a key role in gluconeogenesis in other anaerobic species.

The pyruvate transcarboxylase complex, which initiates the Wood–Werkman cycle through an ATP-independent carboxyl group transfer, is a defining aspect of propionibacteria metabolism. However, three separate enzymes also allow for carbon fixation to produce oxaloacetate and may promote the production of succinate. Two separate PEP carboxykinases were identified: a GTP-dependent variant (4.1.1.32) found only in *P. propionicum* and a recently sequenced polyphosphate-dependent variant [85] found in all propionibacteria studied. Both *P. acidipropionici* and *P. propionicum* contained an additional pyruvate carboxylase (6.4.1.1). The redundancy of carbon fixation mechanisms for PEP and pyruvate coupled with a gluconeogenic malate dehydrogenase (1.1.1.39) found in all strains suggests that there is complex regulation around the pyruvate node.

#### 3.4.5. Respiration

All propionibacteria are able to generate ATP from the Wood–Werkman cycle via a short anaerobic electron transport chain. Electrons are transferred from NADH to intracellular fumarate via an NADH dehydrogenase (1.6.5.3) and fumarate reductase, both of which are linked by membrane potential. Then, the generated proton-motive force is converted to ATP through an ATP synthase. A second copy of the fumarate reductase is thought to act as a succinate dehydrogenase [45]; this theory is further supported by the differential clustering of the two complexes in our pan-genomic analysis (Supplementary file 2, S4) and transcriptomics data for *P. acidipropionici* 4875 where only a single complex was highly expressed (Supplementary file 7, S11). Although it has been demonstrated that NADPH can be coupled to propionate production [61], all species were only found to have a respiration-coupled NAD(P)+ transhydrogenase (1.6.1.2) associated with NADP reduction, suggesting that NADPH couples inefficiently via a core NADPH:quinone oxidoreductase, which is upregulated when *P. acidipropionici* was grown on glycerol (Supplementary file 7, S11).

Propionibacteria differ significantly in their capacity to use different terminal electron acceptors. Despite the genus being viewed as microaerophilic, a cytochrome C linked oxidase, oxidoreductase, and NADH dehydrogenase, allowing full aerobic respiration, were identified in three *Propionibacterium* species; meanwhile, *P*. *freudenreichii* and *P*. *propionicum* had lost most of these subunits. The frameshift in the cytochrome C subunit I protein identified in *P. acidipropionici* 4875 [24] is not present in *P. acidipropionici* 55737, which we believe to possess full aerobic respiratory capabilities. All species contained the cytochrome *bd* oxidase (1.10.3.14) giving propionibacteria a degree of aerotolerance.

**Figure 5:**
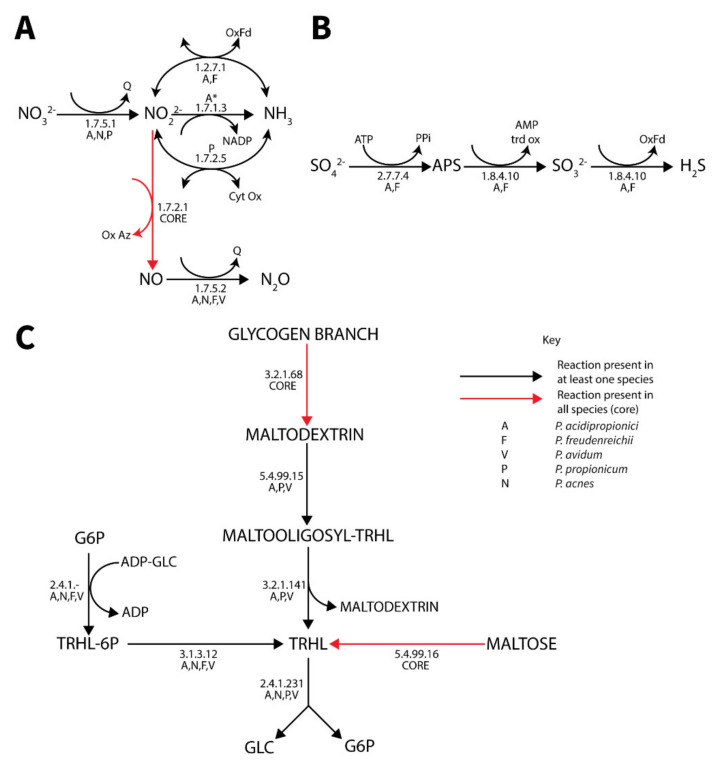
Diagrams of the pan-genomic nitrate (**A**), sulfate (**B**) and trehalose (**C**) metabolism in *Propionibacterium***.** (Black) arrows represent reactions present in at least one species. (Red) arrows represent reaction in all species (core). *Enzyme is present in both *P. acidipropionici* strains but 4875 has a frameshift mutation likely rendering the enzyme non-functional.

A number of alternative electron acceptors were identified. Nitrate metabolism was highly variable between different species but most extensive in *P. acidipropionici* (Figure 5). All species except *P. propionicum* could reduce nitrite to nitrous oxide, while only select species contained nitrate and nitrite reductases. Similar to cytochrome C, the frameshift likely inactivating EC 1.7.1.4 in *P. acidipropionci* 4875 [24] was not present in *P. acidipropionci* 55737. Sulfate reduction to hydrogen sulfide was observed only in the dairy *Propionibacterium* strains (Figure 5). Although a previous attempt to identify the complete pathway for sulfate reduction in *P. acidipropoinici* 4875 was unsuccessful [24], we were able to postulate a complete pathway from the pan-genome matrix that is consistent with the phenotype array data (Supplementary file 5, S9)**.** It was found that an ambiguously annotated enzyme co-annotated with both an adenyl-sulfate reductase functionality (1.8.4.10) and phosphoadenyl-sulfate reductase (1.8.4.8) functionality was most similar to adenyl-sulfate reductase and likely encoded this function. The expanded capability of *P. freudenreichii* and *P. acnes* to utilize alternative electron acceptors was also noted. Both were previously found to contain genes allowing the use of dimethyl sulfoxide as an electron acceptor [24]. We additionally identified homologs of the *fixABCX* genes clustered in the genome and *caiABC* homologs in both genomes, suggesting that these species can catabolize L-carnitine to γ-butyrobetaine, which additionally acts as a terminal electron acceptor.

#### 3.4.6. Polyphosphate Metabolism

Both pyrophosphate and polyphosphate metabolism are distinguishing features of *Propionibacterium* metabolism and are thought to play key roles in central carbon and energy metabolism. All propionibacteria contained both a polyphosphate kinase (2.7.4.1) and polyphosphate glucokinase (2.7.1.63), allowing excess energy to be stored as polyphosphate and used to phosphorylate glucose not transported by the phosphotransferase system, as indicated by a relative upregulation on glucose relative to sucrose in *P. acidipropionici*. This activity is solely polyphosphate dependent in all species studied except *P. propionicum*, which contained an ATP-specific glucokinase. A polyphosphate kinase II was also found in *P. propionicum* and *P. acnes*, which has a tendency for the production of nucleotide triphosphates from diphosphates [86]. We hypothesize this enzyme plays an essential role in the synthesis of nucleotide triphosphates in *P. propionicum*, which lacks the ATP-dependent equivalent. Exopolyphosphatase (3.6.1.11) was also found in all genomes, which controls polyphosphate levels and likely has a regulatory function due to the many signaling roles of polyphosphate [86,87].

Pyrophosphate is associated with a number of reactions in central carbon metabolism. The presence of an inorganic pyrophosphatase (3.6.1.1) and relatively high expression in *P. acidipropionici* (mean ≈ 4550 RPKM, Supplementary file 7, S11) suggests that pyrophosphate is maintained at low levels in the cell. Reactions associated with the metabolite pyrophosphate are likely thermodynamically favorable in the direction of pyrophosphate synthesis, such as the pyrophosphate-dependent fructose 6-phosphate 1-kinase (2.7.1.90) and pyruvate–phosphate dikinase (2.7.9.1) that permit gluconeogenesis. With the exception of *P. freudenreichii*, all genomes contained a pyrophosphate energized proton pump that could build a proton motive force from pyrophosphate-generating enzymes such as the phosphoenolpyruvate carboxytransphosphorylase (4.1.1.38) and pyruvate phosphate dikinase; indeed omics data suggested coupled over-expression with the latter in *P. acidipropionici* (Supplementary file 7, S11).

#### 3.4.7. Trehalose Metabolism

Propionibacteria synthesize trehalose in response to stress [88]. Three trehalose synthesis pathways and one degradation pathway were identified, with synthesis from maltose via the trehalose synthase (5.4.99.16) being the only core functionality across all species (Figure 5). Additionally, the two-step synthesis of trehalose from ADP-glucose and glucose-6-phsophate was identified in all strains except *P. propionicum*, while a final synthesis pathway linking glycogen metabolism to trehalose synthesis was identified in *P. acidipropionici*, *P. propionicum,* and *P. avidum*. While all strains except *P. freudenreichii* could degrade trehalose via trehalose phosphorylase, it has been suggested that trehalose synthase plays a degradation role in this species [88]. It is worth noting that *P. acidipropionici* and *P. avidum* not only contain all trehalose metabolic functionalities of *Propionibacterium* but also contain multiple copies of several genes including trehydrolase (3.2.1.141) and trehalose phosphorylase (2.4.1.231), while *P. acidipropionici* additionally contains glycine betaine synthesis capability. This expanded osmoprotectant metabolism in *P. acidipropionici* suggests a potential role of osmoprotectants in alleviating acid stress to enable the high acid-producing phenotype typical of the species. These findings are supported by a trehalose over-expression study in *P. acidipropionici* 4875 [16].

#### 3.4.8. Amino Acid Biosynthesis and Catabolism

Amino acid metabolism in propionibacteria is particularly relevant to industrial applications of propionibacteria given that it contributes to the formation of flavor compounds in cheese; it has been reported to play key roles in stress tolerance, and it can influence the distribution of fermentation products. The only propiogenic amino acid catabolic pathways identified were for threonine in all species and methionine in *P. acidipropionici* 55737 only, which apart from a methionine γ lyase was otherwise identical in amino acid metabolism to strain 4875. Branched-chain amino acids were only partially degraded to fatty acid and alcohol derivatives, consistent with experimental evidence [89] and the phenotype array data collected for *P. acidipropionici* 4875 where they could serve as nitrogen but not carbon sources. While *P. avidum* is known to possess D-isomer 2-hydroxyacid dehydrogenase activity, a candidate gene encoding this functionality in *P. avidum* but absent in other tested strains could not be identified [90].

**Figure 6:**
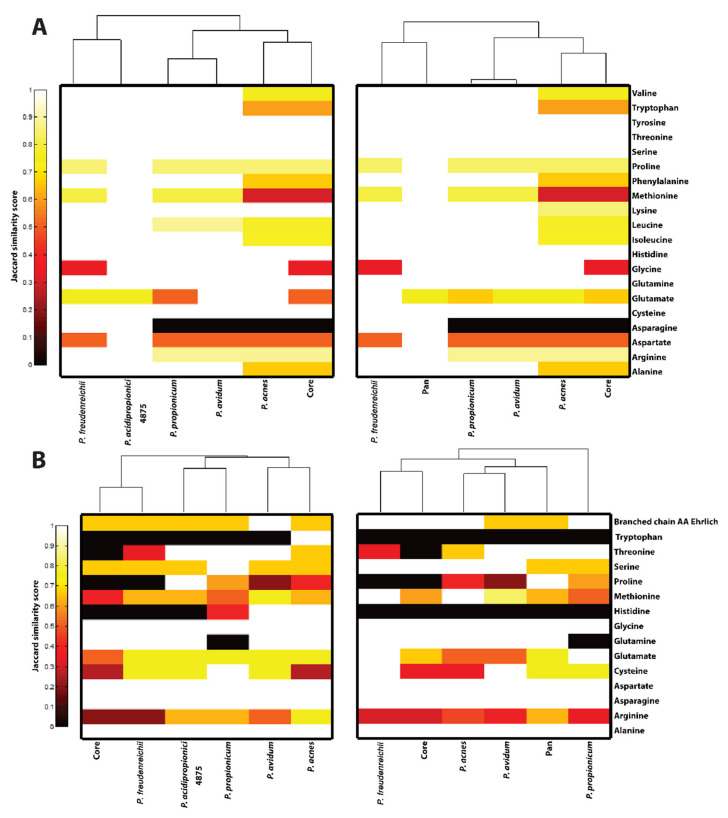
Heat map and clustering of amino acid biosynthesis (**A**) and degradation (**B**) pathways using the Jaccard similarity metric. In this metric, 0 represents no reactions in common, and 1 represents all reactions shared. Plots to the left are referenced to the pan-reactome (distances represent the proportion of all reactions of the pan-genome ascribed to that pathway present in the organism), while plots to the right represent the same information referenced to *P. acidipropionici* 4875. Commensal strains are observed to have a reduced set of biosynthetic capabilities for amino acids and cluster out from the dairy strains. Extensive variability is observed in the arginine, methionine, and proline degradation pathways. The reductive arginine degradation pathway is observed in *P. acidipropionici*, while the oxidative pathway is observed in commensal species, possibly reflecting adaptation to aerobic conditions. Histidine and tryptophan degradation capabilities are observed only in selected commensal strains.

The ability for strains to synthesize and degrade amino acids will have an influence on the redox balance of the cell and consequently, the fermentation products. Differences in the metabolic potential for amino acid synthesis or degradation between strains were quantified using the Jaccard similarity coefficient and visualized as clustergrams (Figure 6). Examination of the biosynthetic pathways reveals distinct clustering of commensal and dairy bacteria. Interestingly, commensal organisms were found to have reduced diversity in their biosynthetic pathways and a wider range of degradative capabilities, particularly in comparison to *P. freudenreichii*. These findings likely reflect the greater reliance of proteolytic activity as a source of carbon and energy among commensal propionibacteria given their nutrient-scarce microenvironment [10]. Although representative species for all four genera that compose the former *Propionibacterium* genus were considered in this analysis, the analysis needs to be expanded further to encompass newly available sequenced species [91,92,93] before it can be generalized more broadly. Only *P. acnes* was detected as auxotrophic for some amino acids.

Acid tolerance catabolic activities were also variable between species. All species contained L-asparaginase, which has been associated with acid tolerance in *P. freudenreichii* [94]. The arginine deiminase pathway and associated antiporter were identified in *P. avidum* and *P. acnes*, while the glutamate decarboxylase and associated antiporter were found in *P. freudenreichii*. Both *P. acidipropionici* strains contained both mechanisms without antiporters, suggesting they were less specialized to a particular acid tolerance mechanism. However, these more traditional acid tolerance mechanisms associated with Gram-positive bacteria [95] are unlikely to reflect the true extent of possible acid tolerance amino acid catabolic activity. Phenotype array data suggest that amino acid deamination may play a key role; *P. acidipropionici* could utilize all 20 common amino acids as a sole nitrogen source but less than half as a carbon source. This functionality is not captured in the current model of *P. acidipropionici* because no genes associated with these functionalities were detected, as is the suggested degradation capability of phenylalanine where no homologues could be found to any known catabolic pathways, suggesting the potential presence of a novel catabolic pathway.

## 4. Conclusions

We have developed six genome-scale metabolic reconstructions spanning five propionibacteria to aid in silico investigation into the poorly understood *Propionibacterium* metabolism. A pan-genomic guided metabolic reconstruction strategy was developed to help overcome bioinformatic challenges associated with these non-model, high GC-content organisms. The parallel curation of several related species leads to a significantly improved annotation for all reconstructions.

Comparison of the resulting reconstructions revealed that central carbon metabolism was found to be highly variable between species across all the key nodes surveyed in this study. This is especially true for acetate production for which no conserved pathway existed across the five *Propionibacterium* species despite being the second major fermentation product. This variability was even observed at a strain level of analysis and was further exemplified in amino acid metabolism, where it was suggested that commensal organisms have acquired a broader range of degradative capabilities but a reduced set of biosynthetic capabilities compared to their dairy counterparts.

By exploring the functional capacity of *Propionibacterium* metabolism through the metabolic reconstructions, we also identified clues for the evolution of the Wood–Werkman cycle and were able to propose a novel ferredoxin-linked energy-conserving strategy and putative gene candidates. This work lays the foundations for further advancement in the understanding of *Propionibacterium* metabolism, and we expect the reconstructions presented here to be instrumental in the further investigation of propionibacteria, such as designing metabolic engineering strategies to enhance the potential for propionibacteria to produce industrially relevant chemicals or to better understand the relationship between propionibacteria and human health through metabolic modeling.

## Figures and Tables

**Table 1:** List of strains with complete genomes used in the pan-genomic study and model reconstruction.

Species	Strain	Accession	Reference	Metabolic Reconstruction
Dairy propionibacteria (*Acidipropionibacterium*, *Propioinibacterium*)				
*A. Acidipropionici*	ATCC 4875	NC_019395.1	[24]	Yes
	ATCC 55737	NZ_CP014352.1	[35]	Yes
*P. freudenreichii*	CIRM-BIA1	NZ_CP010341.1	[23]	Yes
Commensal propionibacteria (*Cutibacterium*, *Pseudopropionibacterium*)				
*C. avidum*	44067	NC_021064.1	[36]	Yes
*P. propionicum*	F0230a	NC_018142.1	[37,38]	Yes
*C. acnes*	KPA171202	NC_006085.1	[39]	No
	6609	NC_017535.1	[40]	Yes
	TypeIA2 P.acn17	NC_016512.1	[41]	No
	TypeIA2 P.acn31	NC_016511.1	[41]	No
	TypeIA2 P.acn33	NC_016516.1	[41]	No
	ATCC 11828	NC_017550.1	[42]	No
	C1	NC_018707.1	[43]	No
	HL096PA1	NC_021085.1	[44]	No
	SK137	NC_014039.1	[37,38]	No
	266	NC_017534.1	[45]	No
	hdn-1	NZ_CP006032.1	(Nagy et al., unpublished)	No

**Table 2:** List of plasmids and primers.

Plasmids		Reference
pRGO1	Plasmid from *P. acidipropionici*	Kiatpapan [62]
pBRPprp_gfpuv	PBR322-based plasmid. gfpUV expression under the control of prpR promoter. AmpR	Lab collection
pPAC_Cas9	pRGO1-derived plasmid harboring optimized Cas9. EryR. ApraR	This work
pCas9_nifJ	pPAC_Ca9-derived plasmid. Contains gRNA region for knockout of nifJ	This work
**Primers**		
ackUP-HA_fwd	tttttaagcttcccgTCTCGCCGCTACCGCGCTTG	
ackUP-HA_rev	ggatagcgtcgccgtACGCCGCTGGCCGGCCTG	
ack-gfpUV_fwd	gccggccagcggcgtACGGCGACGCTATCCCCA	
ack-gfpUV_rev	tcggcgagctcaaccTTATTATTTGTAGAGCTCATCCATGCCATG	
ack-DW-HA_fwd	ctctacaaataataaGGTTGAGCTCGCCGAGGTCG	
ack-DW-HA_rev	aattggagctccaccgcggtggcggccgctCGGAGAACCCGGTGGCCG	
ack conf_fwd	CCGAGCATTCCCGAGTTC	
ack conf_rev	CTTCGACACCGCCTTCTTC	
20 nt region	ccgccgggcgcaccaacctg**TGG** (PAM region shown in bold not included in gRNA scaffold)	

**Table 3:** Summary of extent of manual curation across the different models. GPR: gene–protein–reaction associations.

Model	*P. acidi.* 4875	*P. acidi.* 55737	*P. freud*	*P. avidum*	*P. prop*	*P. acnes*
Reactions added	250	239	219	237	213	226
Transporters added	117	121	121	131	131	127
Reactions removed	26	22	29	21	22	19
GPR altered	139	141	107	119	111	122
Total reactions	1050	1056	933	962	977	952
Total transporters	242	241	217	229	218	231
Reactions without GPR	55	45	49	56	49	45
Transporters without GPR	103	103	109	112	115	109

Table 3 summarizes key aspects of the manual curation performed of the draft reconstructions such as the addition or removal of metabolic reactions and metabolite transporters, alterations to the gene–protein–reaction associations (GPR), and the final model size and GPR coverage.

**Table 4:** Summary of extent of manual curation across the database.

Database Modifications	
Reaction directionality changes	116
New reactions added to database	105
New metabolites added to database	28

Table 4 summarizes the extent of manual curation that occurred on information from the KBase database where the model was sourced from: namely, corrections to the thermodynamic bounds of reactions, corrections of compounds participating in a reaction (generally preferred over generating entirely new reactions), and the additional reactions to describe biochemistry absent from the database.

**Table 5:** Minimal media requirements as determined during the reconstruction process.

Nutrient Requirement		*P. acidipropionici*	*P. freudenreichii*	*P. avidum*	*P. propionicum*	*P. acnes*
**Vitamins**	Biotin	Biotin	Biotin	Pimelate	Biotin	Pimelate
	Pantothenate	Pantothenate	Pantothenate	Pantothenate	Pantothenate	Pantothenate
	NAD				Nicotinate	Nicotinate
	Thiamin			Thiamin	Thiamin	Thiamin
	Riboflavin				Riboflavin	
	Pyridoxal				Pyridoxal	
	Folate				*p*-aminobenzoate	
**Amino acids**	Methionine					Methionine
	Isoleucine					Isoleucine
	Leucine					Valine
	Valine					Valine
	Tryptophan					Tryptophan
	Phenylalanine					Phenylalanine
**Others**	CTP				CMP	

Table 5 lists the absolute minimal nutritional additives that should be added to a basic media containing salts and a source of carbon, nitrogen, phosphate and sulfur to allow growth of the listed propionibacteria, as predicted from the metabolic reconstructions.

## Supplementary Materials

The following are available online at https://www.mdpi.com/2073-4425/11/10/1115/s1. Supplementary file 1, S1: Limits of applying the Pan-genome approach to partial genomes. S2: Gene caller trials. S3: Depictions of the robust core and pan-genome and metabolic functionalities excluded by this approach. S5: Estimates of the core and pan genome size of the Propionibacterium genus based on 5 species. S8: Manual curation guide for a RAST-based reconstruction. S12: Transcriptomics visualisation for relevant genes. Supplementary file 2, S4: Pan-genomic matrix representations based on orthologous clusters and partial and full functional compactions; used to guide manual curation of the model. Supplementary file 3, S6: Consensus biomass equation reconstruction. Supplementary file 4, S7: Final Pan-GEM model and original GEMs resulting from the reconstruction pipeline, corresponding xml and mat files and associated GenBank files in a zipped folder. Supplementary file 5, S9: BIOLOG data for *P. acidipropionici* 4875 used to guide gap-filling work and manual curation. Supplementary file 6, S10: Definition and analysis for presence/absence of the amino acid catabolic and anabolic pathways in various species and computation of the Jaccard distances. Supplementary file 7, S11: Phenotype data collected from *P. acidipropionici* 4875 grown on three different carbon sources, including extracellular metabolite concentrations, proteomics and metabolomics data.
