# Supplementary material for: A Pan-Genome Guided Metabolic Network Reconstruction of Five Propionibacterium Species Reveals Extensive Metabolic Diversity
Source: Genes (Basel). 2020 Sep 23;11(10):1115. doi: 10.3390/genes11101115 (PMC7650540; doi:10.3390/genes11101115)
Supplement: Supplementary file 1 [file genes-11-01115-s001.zip › Final version of supplementary material/Supplementary file 1 S1-3,S5,S8,S12.docx]

**Additional file 1**

Table of Contents

[S1: Limits of applying the Pan-genome approach to partial genomes 2](#_Toc46917617)

[S2: Gene caller trials 5](#_Toc46917618)

[S3: Depictions of the robust core and pan-genome and metabolic functionalities excluded by this approach 11](#_Toc46917619)

[S5: Estimates of the core and pan genome size of the *Propionibacterium* genus based on 5 species 13](#_Toc46917620)

[S8: Manual curation guide for a RAST-based reconstruction 18](#_Toc46917621)

[S12: Transcriptomics visualisation for relevant genes 26](#_Toc46917622)

[References 27](#_Toc46917623)

# S1: Limits of applying the Pan-genome approach to partial genomes

We performed a pan-genomic analysis using the Get_homologues package [1] and a pan-GEM analysis by generating draft GEMs using the RAST pipeline [2–4] on increasingly incomplete genomes to assess its susceptibility to contigs. Closed genomes were obtained for *P. acidipropionici* 4875 and 55737 strains from NCBI and partial *P. acidipropionici* 55737 genomes were obtained through Illumina sequencing for *P. acidipropionici* ATCC 55737. This high-throughput sequence data was analysed through Velvet [5] resulting in 2038 contigs and closed further to 737 contigs using paired-end reads with the SPAdes [6] algorithm to generate two partial genomes. The pan-genome and pan-GEM analyses were performed by following the change in size of the core and pan protein clusters or reactions respectively on sequential addition of the incomplete genomes to the two closed genomes. This analysis allows comparison of the inter-strain variability through the inclusion of the two closed genomes and the variability attributed to incomplete genomes.

| Analysis | *P. acid* 4875 | *P. acid* 55737 PacBio | *P. acid* 55737 SPAdes | *P. acid* 55737 Velvet |
| --- | --- | --- | --- | --- |
| No. contigs | 1 | 1 | 737 | 2038 |
| Core genome size | 3267 | 2786 | 2306 | 1856 |
| Core reduction | 0 | 481 | 480 | 450 |
| Pan genome size | 3267 | 3600 | 3743 | 3866 |
| Pan increase | 0 | 333 | 143 | 123 |
| Core GEM size | 970 | 950 | 933 | 885 |
| Core GEM reduction | 0 | 20 | 16 | 48 |
| Pan GEM size | 970 | 990 | 994 | 995 |
| Pan GEM increase | 0 | 20 | 4 | 1 |
| Core GEM + gap fill size | 1066 | 1051 | 1032 | 986 |
| Core GEM + gap fill reduction | 0 | 15 | 19 | 46 |
| Pan GEM + gap fill size | 1066 | 1094 | 1103 | 1108 |
| Pan GEM + gap fill increase | 0 | 28 | 9 | 5 |

Table 1: Analysis of the influence of adding partial sequence data to genomic comparison type analyses. Comparisons have been performed between two complete organisms of the same species and consecutively by adding increasingly incomplete genomes to the analysis from different sequencing technologies and assemblers such that the variability between two strains can be compared to that between partial sequence data for the same strain. Both classical pan-genomic type analyses and a more novel pan-genome scale model (GEM) type analysis have been performed where the sizes refer to the number of protein clusters and the number of metabolic functionalities (reactions) in the models respectively.

The pan-genome analysis could be seen to be overly sensitive to contigs. By following the reduction in core genome size it can be seen that the introduction of 737 and 2028 contig genomes has a similar outcome as introducing a new strain to the analysis each time. The pan-genome was also influenced but was less sensitive, the increase in pan-genome size was only about 40% of the increase attributed to the addition of a new strain. This corresponds to approximately a loss of a core gene or protein every 2.2 contigs introduced or a gain in pan-genome size of 1 per 7.9 contigs introduced.

Surprisingly, the pan-GEM analysis performed on metabolic models from the RAST pipeline showed an even greater proportional drop in the core-reactome by the introduction of contigs than to the introduction of a new strain. The introduction of 1300 contigs between the SPAde and Velvet based models reduced the core reactome by 48 reactions, or about 2.4 times the inter-strain variability. The pan-reactome size showed much less variability; the total increase in reactome attributed to the introduction of contigs was only 25% of the total increase attributed to the introduction of a different strain. Combining the approach with automated gap-filling on a complete media with the general biomass equation from the RAST framework made little difference to the reduction of the core-genome. However, it should be noted that by only considering the influence of metabolic genes and due to redundancies in the genome, the reconstruction of the metabolic models appeared much less sensitive to the introduction of contigs. The reduction in core reactome was only about 2 and 5% respectively with the introduction of the SPADES and Velvet models, compared to the reduction of 17 and 24% of the core-genome. The real benefit from the pen-GEM approach is that essential reactions lost can be caught by automated gap-filling procedures and missing metabolic genes may be easily identified by the presence of blocked reactions or partial pathways in metabolic models. On the basis of these results, we suggest that the pan-GEM approach is less sensitive to contigs and is therefore more useful when comparing high-throughput sequencing data typical of metagenomic investigations.

# S2: Gene caller trials

Gene callers were assessed and compared using three different methods. First, gene callers were assessed by the number of essential gaps for biosynthesis that were identified for the raw model. Secondly, the differentially called reactions were analysed between the gene callers to identify metabolic functionalities that were not incorporated into the reconstruction because either the gene was not called or had a variable start and/or stop site and was not specifically associated with a relevant metabolic functionality in the initial reconstruction. Finally, the gene callers were compared directly by analysing the number of intergenic peaks from transcriptomics data generated from complex media fermentations using three different carbon sources. Seven different gene-caller annotations were tested:

- NCBI: the original annotation of the *Propionibacterium acidipropionici* 4875 available from NCBI which used Glimmer trained on *P. acnes*.
- GeneMark: the GeneMark annotation of *Propionibacterium acidipropionici* 4875 downloaded from NCBI. The highest scoring gene annotations were used to select the gene end points.
- GeneMarkHMM: the GeneMarkHMM annotation of *Propionibacterium acidipropionici* 4875 downloaded from NCBI.
- Glimmer: the Glimmer annotation sourced from NCBI.
- Prodigal: an annotation generated in-house using Prodigal software
- RAST: annotation using the default Glimmer based gene caller of RAST which is iteratively trained on new annotations
- Dual: The combination of the RAST implementations of Glimmer and Prodigal with overlapping genes reconciled using the functionality of RASTtk.

1. *Studying the efficacy of gene calling algorithms using gap-filling or missing reactions as a metric*

The 7 gene calling algorithms were compared on their inability to call essential genes required for biomass generation under two media conditions; a complete media and an experimentally derived minimal media with glucose as a carbon source. Analysis was restricted to non-universal gap-fills and was performed on raw model outputs from RAST without any manual curation efforts. Gap-filling solutions required reconciliation before comparison due to the ability of the algorithm to choose alternative solutions stochastically and occasionally, non-minimal solutions through the weighted *l_1_*-norm (minimisation of flux) gap-fill formulation.

After reconciliation, differences between the algorithms were minor. The complete media has a single gap associated with isoprenoid biosynthesis that was missed by the Glimmer based algorithms; Glimmer, Dual, RAST and NCBI. Two further gaps were identified in minimal media associated with folate biosynthesis by GeneMark and methionine biosynthesis by the Glimmer and Dual algorithms.

| **Gene Caller** | NCBI | RAST | Dual | Glimmer | Prodigal | GeneMark | GeneMarkHMM |
| --- | --- | --- | --- | --- | --- | --- | --- |
| **Metabolic gaps** | 1 | 1 | 2 | 2 | 0 | 1 | 0 |

Table 1: Summary of non-universal metabolic gaps resulting from different gene-calling algorithms

1. *Comparison by analysing differentially called genes associated with model reactions*

While the gap-fill approach is restricted to key pathways associated with growth, comparison of all differentially called reactions may give a clearer depiction of differential gene prediction between algorithms. For this work, the NCBI annotation was used as a reference. Differentially called genes were analysed for their consensus and subsequently validated. As some genes encode multiple metabolic reactions, results were reconciled into gene based solutions. Interestingly, the dual algorithm predicted several non-consensus reactions which were not genes missed by other algorithms but non-specific annotations which became specific and therefore linked to a reaction when gene boundaries were reconciled between Glimmer and Prodigal predictions. These cases were analysed on an individual basis.

|  | RAST | Dual | Glim | Prod | Gene  Mark | Gene  Mark  HMM |
| --- | --- | --- | --- | --- | --- | --- |
| **Consensus reactions added to NCBI** | Genes present | | | | | |
| L-Lactaldehyde[c0] + NADP[c0] <=> H+[c0] + 2-Oxopropanal[c0] + NADPH[c0] |  |  |  |  |  |  |
| Nicotinamide[c0] + H2O[c0] => NH3[c0] + Niacin[c0] |  |  |  |  |  |  |
| H+[c0] + 1-Hydroxy-2-methyl-2-butenyl 4-diphosphate[c0] + NADPH[c0] => Isopentenyldiphosphate[c0] + NADP[c0] + H2O[c0] |  |  |  |  |  |  |
| NADP[c0] + H2O[c0] + DMAPP[c0] <= H+[c0] + 1-Hydroxy-2-methyl-2-butenyl 4-diphosphate[c0] + NADPH[c0] |  |  |  |  |  |  |
| H+[c0] + NADH[c0] + 1-Hydroxy-2-methyl-2-butenyl 4-diphosphate[c0] => H2O[c0] + NAD[c0] + DMAPP[c0] |  |  |  |  |  |  |
| H+[c0] + NADH[c0] + 1-Hydroxy-2-methyl-2-butenyl 4-diphosphate[c0] => Isopentenyldiphosphate[c0] + H2O[c0] + NAD[c0] |  |  |  |  |  |  |
| **Total genes added** | 2 | 2 | 3 | 3 | 2 | 3 |
| **Consensus missing reactions vs. NCBI** | Genes absent | | | | | |
| H2O[c0] + L-Arginine[c0] => Urea[c0] + Ornithine[c0] |  |  |  |  |  |  |
| ATP[c0] + Glycerate[c0] <=> 3-Phosphoglycerate[c0] + ADP[c0] |  |  |  |  |  |  |
| PPi[c0] + Phosphoribosyl-ATP[c0] <= ATP[c0] + PRPP[c0] |  |  |  |  |  |  |
| NADP[c0] + 5-Methyltetrahydrofolate[c0] <=> H+[c0] + 5-10-Methylenetetrahydrofolate[c0] + NADPH[c0] |  |  |  |  |  |  |
| 5-Methyltetrahydrofolate[c0] + NAD[c0] <=> H+[c0] + NADH[c0] + 5-10-Methylenetetrahydrofolate[c0] |  |  |  |  |  |  |
| Dihydroneopterin[c0] <=> 6-hydroxymethyl dihydropterin[c0] + Glycolaldehyde[c0] |  |  |  |  |  |  |
| ATP[c0] + Adenosyl cobinamide[c0] <=> H+[c0] + Adenosyl cobinamide phosphate[c0] + ADP[c0] |  |  |  |  |  |  |
| H+[c0] + Adenosyl cobinamide phosphate[c0] + GTP[c0] <=> Adenosylcobinamide-GDP[c0] + PPi[c0] |  |  |  |  |  |  |
| Adenosyl cobinamide[c0] + GTP[c0] <=> H+[c0] + Adenosyl cobinamide phosphate[c0] + GDP[c0] |  |  |  |  |  |  |
| **Total genes missed** | 3 | 3 | 1 | 1 | 0 | 0 |

Table 2: Reactions ascribed to genes in models resulting from different gene calling algorithms.

The three reactions non-consensus reactions annotated only in the dual algorithm are as follows:

1. Cobalt-precorrin-6A reductase (EC 1.3.1.54)

The gene was called in all gene calling algorithms. In most cases it had exactly the same starting position and length, although the length did vary in the GeneMark annotation (where the gene end position was specified choosing the highest scoring position). Despite this, the reaction was only present in the dual model. The gene is likely correct given that it sits in a region of genes encoding related functionalities.

1. Trehalose phosphatase

This reaction was absent in all other models but the gene was called and assigned as a general phosphatase. In some but not all cases this may have been due to the starting position of the gene shifting, although in cases where the start point and length of gene are identical it is unclear why the RAST algorithm differentially annotates the gene one way or the other. Because of the genomic context of this gene, which sits beside trehalose biosynthesis genes, it is likely that annotation is correct.

1. Ubiquinone biosynthesis monooxygenase

Because other algorithms predict that this reaction is associated with an ABC type protein and falls into a genomic region heavily populated by transporter associated proteins there is no genomic evidence to suggest that the functionality assigned only to the dual algorithm is the correct functionality in this case.

1. *Comparison of gene callers by analysing numbers of intergenic peaks from transcriptomic data*

For this analysis transcriptomics data from complex media fermentations with either glucose, glycerol or sucrose were performed in duplicate. Peaks were mapped to the genome and counted for the number of times they occurred completely outside of annotated genes (partial overlaps were excluded). These numbers were averaged between both replicates and are presented below.

Figure 1: Number of intergenic peaks for each gene caller on three different carbon sources.

**Outcome**

Gene callers were ranked by each of the three criteria assessed above. It is clear that the number of intergenic peaks does not correlate strongly with the number of functional gaps or the number of reactions called, indicating metabolically functional genes are not representative of the overall gene calling efficiency. Functional metrics were difficult; while gap-filling was not as informative as the differential calling of reaction encoding genes, the pipeline appeared to decide to specifically annotate genes with a given function or not based on slight shifts in gene boundaries or unknown variables. While there are no consistently strongly performing algorithms, Prodigal and GeneMarkHMM performed well generally, corresponding to the two algorithms that incorporated unsupervised learning features, while Glimmer performed well in the intragenic peak analysis. Importantly, we show that using a combination of gene callers can improve the initial GEM annotation by overcoming some quirks in the automated annotation pipelines.

| **Rank** | **Ranked by gaps** | **Ranked by reactions** | **Ranked by intragenic peaks** |
| --- | --- | --- | --- |
| 1 | GeneMarkHMM, Prodigal | GeneMarkHMM | NCBI |
| 2 | GeneMark, NCBI, RAST | GeneMark, Prodigal, Glimmer | Glimmer |
| 3 | Glimmer, Dual | Dual | GeneMarkHMM, Prodigal |
| 4 |  | NCBI | Dual |
| 5 |  | RAST | RAST |
| 6 |  |  | GeneMark |

Table 3: Gene callers ranked by various criteria demonstrate that there is no strong correlation between the number of intergenic peaks and the number of reactions missing from the annotation due to missed gene calls

# S3: Depictions of the robust core and pan-genome and metabolic functionalities excluded by this approach

Depictions of the robust core and pan-genome generated through the Get_homologues software and lists of metabolic functionalities that clustered uniquely and were therefore dropped from the analysis, highlighting issues encountered when trying to extract functional meaning from a traditional pan-genomic approach.


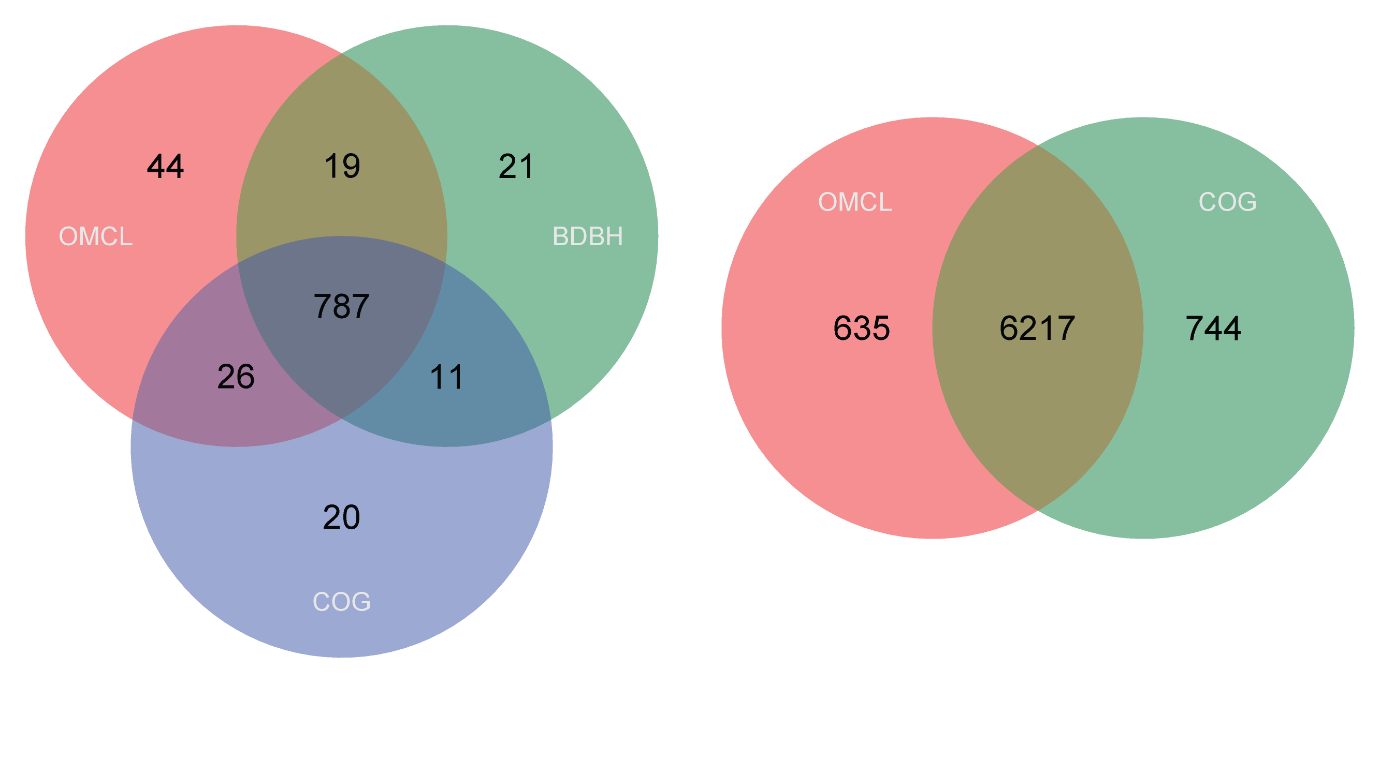


Figure 1: Representations of the robust core and pan genomes generated by Get_homologues showing about 14 and 18% of the clusters generated are not consistent and are therefore removed from the analysis.

| L-ribulose-5-phosphate_4-epimerase_-EC_5.1.3.4- |
| --- |
| 2-4-dienoyl-CoA_reductase_-NADPH-_-EC_1.3.1.34- |
| Methylmalonyl-CoA-Pyruvate_transcarboxylase_12S_subunit_-EC_2.1.3.1- |
| Membrane_alanine_aminopeptidase_N_-EC_3.4.11.2- |
| Beta-galactosidase_-EC_3.2.1.23- |
| Alpha-glucosidase_-EC_3.2.1.20- |
| Dihydroorotate_dehydrogenase-_catalytic_subunit_-EC_1.3.3.1- |
| Cysteine_synthase_-EC_2.5.1.47- |
| NAD-dependent_malic_enzyme_-EC_1.1.1.38- |
| Thiamin-phosphate_pyrophosphorylase_-EC_2.5.1.3- |
| Lead-_cadmium-_zinc_and_mercury_transporting_ATPase_-EC_3.6.3.3-_-EC_3.6.3.5--_Copper-translocating_P-type_ATPase_-EC_3.6.3.4- |
| Dihydroxyacetone_kinase-_ATP-dependent_-EC_2.7.1.29- |
| Exodeoxyribonuclease_VII_small_subunit_-EC_3.1.11.6- |
| Thiosulfate_sulfurtransferase-_rhodanese_-EC_2.8.1.1- |
| Cell_division_protein_FtsH_-EC_3.4.24.-- |
| Copper-containing_nitrite_reductase_-EC_1.7.2.1- |
| Adenosine_deaminase_-EC_3.5.4.4- |
| Galactokinase_-EC_2.7.1.6- |

Table 1: List of clusters associated with EC numbers that were excluded from the core analysis due to differential clustering of the protein translated sequences. Many more functions are excluded from the pan analysis. Notably these contain key metabolic enzymes including the pyruvate oxidase and the 12S subunit of the transcarboxylase from the Wood-Werkman cycle. Such exclusions obscure important details in the functional differences between different species as well as functional capabilities of the entire genus.

#
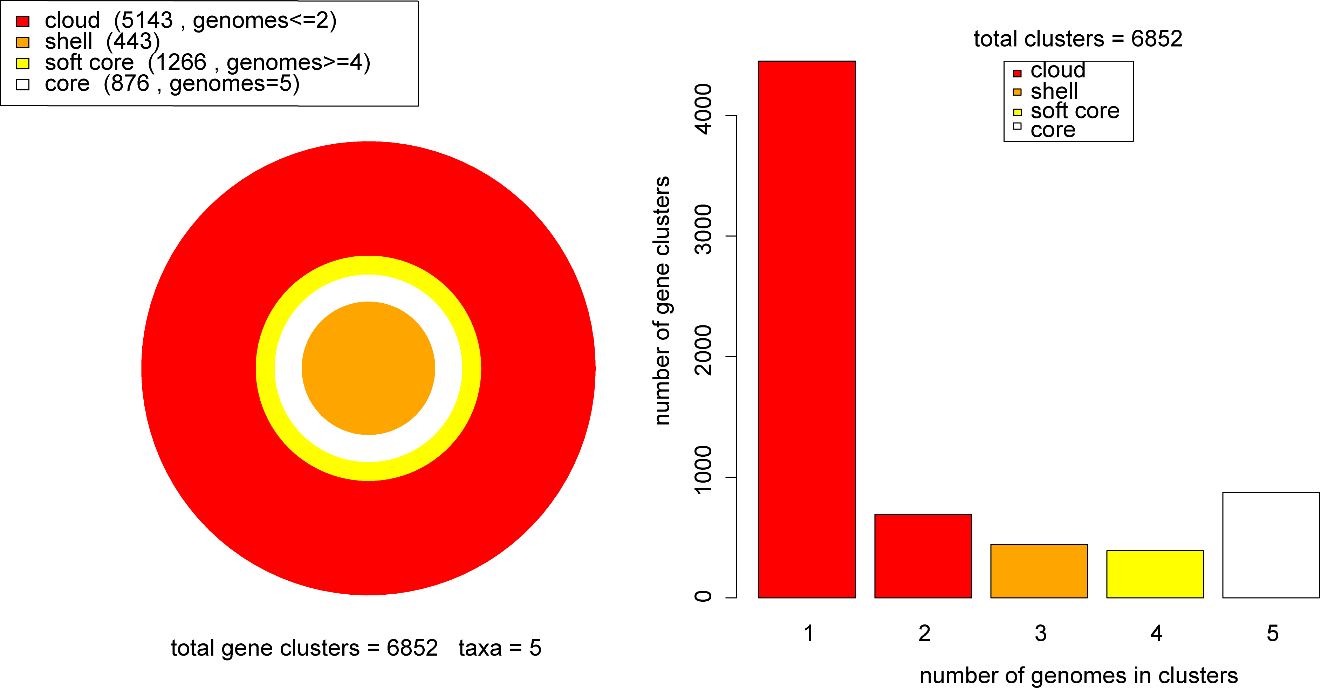
S5: Estimates of the core and pan genome size of the *Propionibacterium* genus based on 5 species, obtained using the Get_homologues package as described in Methods
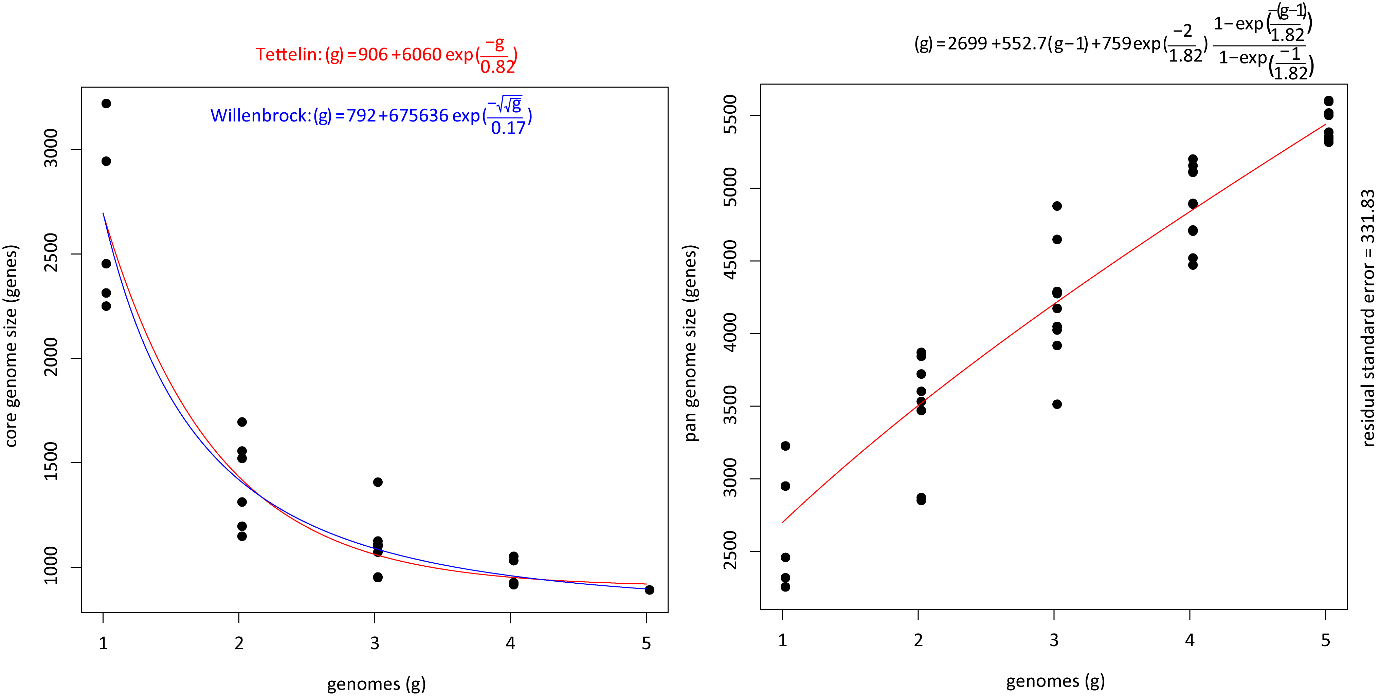


Figure 1: Estimates of the core genome size using the Tettelin and Willenbrock algorithms and pan-genome size estimated using the Tettelin algorithm, based on OMCL clusters generated using the translated protein sequence. A range of 792-906 genes are predicted to be conserved between all Propionibacterium while 553 new genes are expected to be identified with each new species sequenced.

Figure 2: Representations of the pan-genome illustrating a high degree of variability between different Propionibacterium.

*
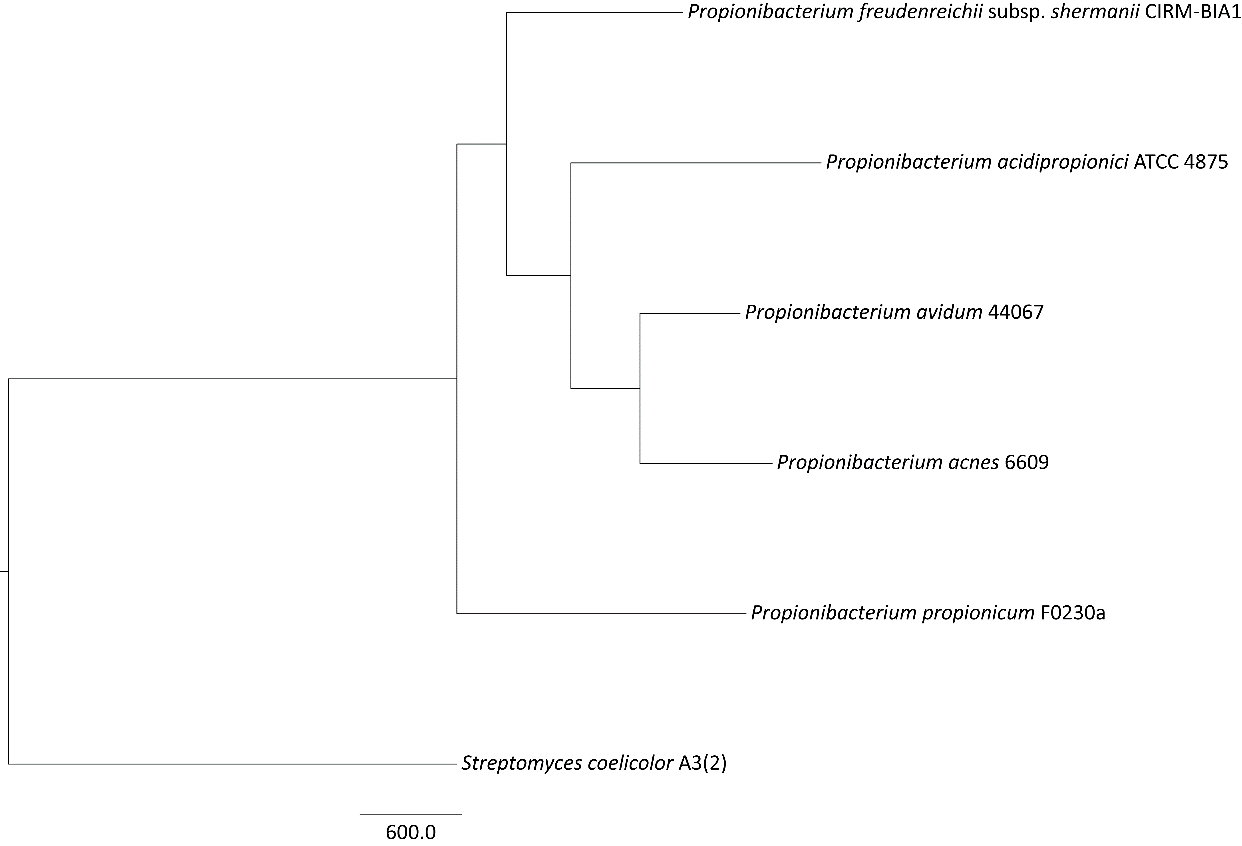
*

*Figure 3:* Phylogenetic tree of closed *Propionibacterium* genomes of representative strains from each species, outgroup rooted using *Streptomyces coelicolor*. Substantial genetic differences can be seen between both species and between strains of the same species.


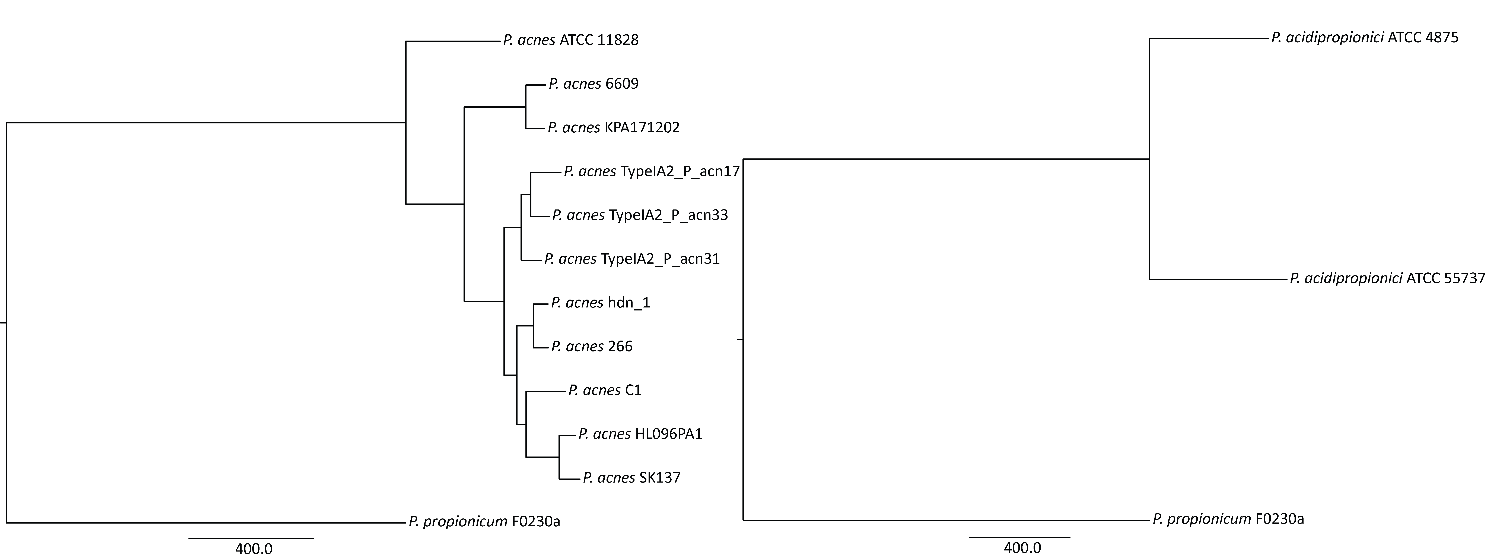


Figure 4: Phylogenetic trees of P. acnes and P. acidipropionici outgroup rooted using P. propionicum. Evolutionary distance between strains is approximated to be about one third of the distance between species in each case.


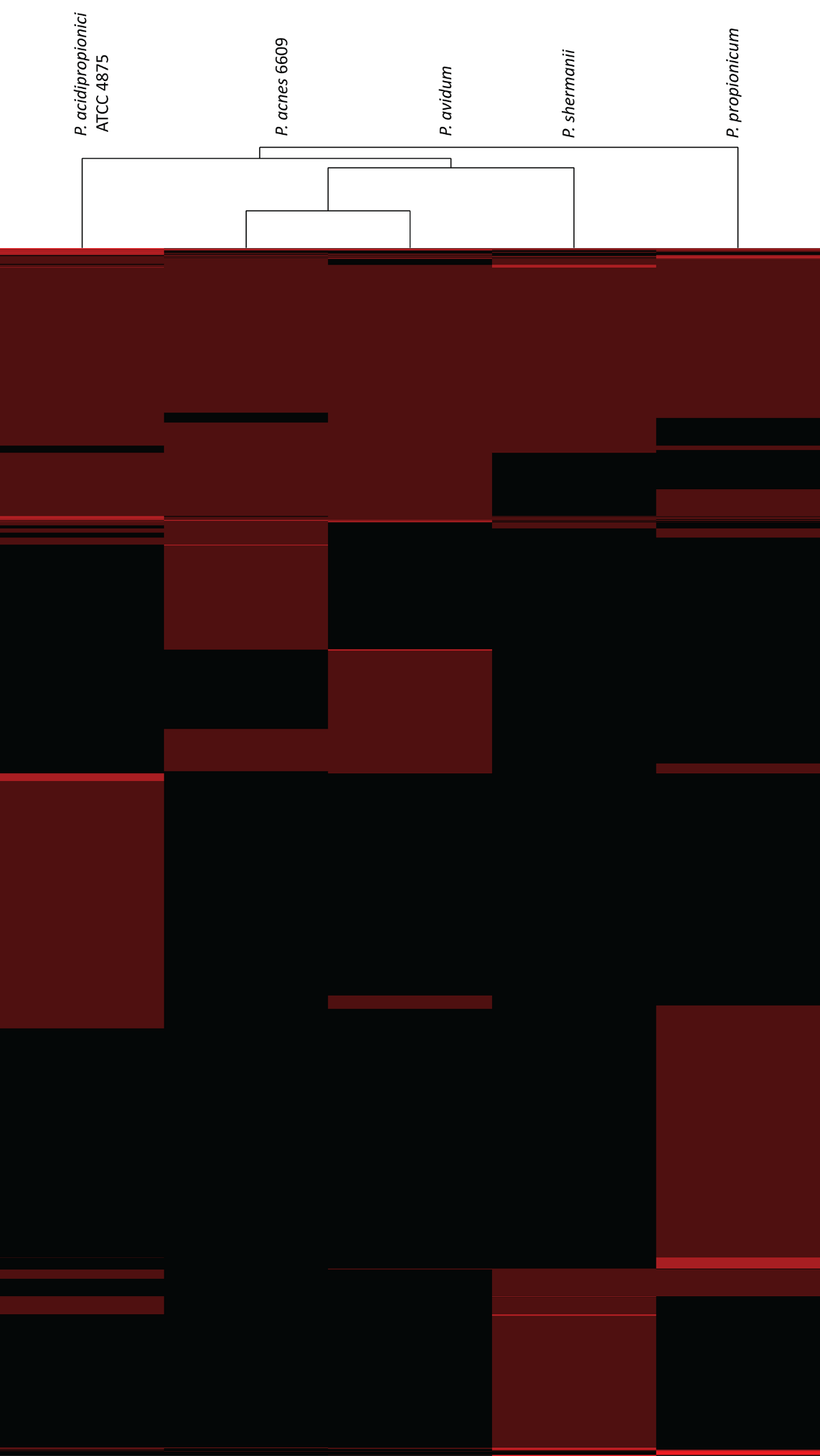


Figure 5: Heat map of orthologous clusters identified in the pan-genomic analysis, depicting considerable differences in the genomes between different species.


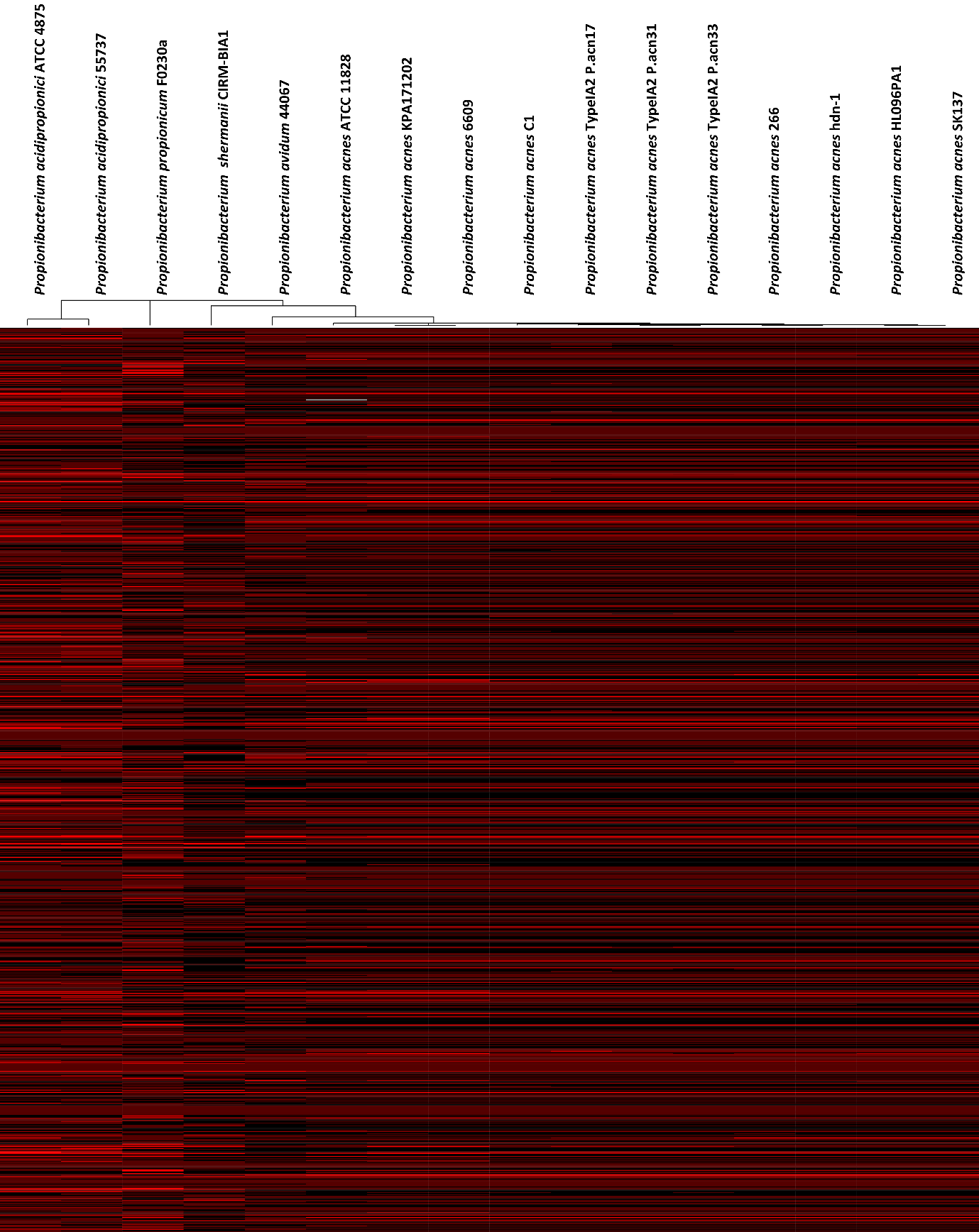


*Figure 6:* Heat map of the functional characteristics between different *Propionibacterium* species.

# S8: Manual curation guide for a RAST-based reconstruction

When performing manual curation, it is important to understand the advantages and limitations of the automated annotation pipeline underpinning the initial reconstruction. Here we attempt to address the scope and type of manual annotation work required to achieve a quality reconstruction based on the RAST annotation pipeline that underpins KBase and ModelSEED. The RAST pipeline is designed to give a high quality initial annotation based on manually curated FIGfams, which inherently leads to a smaller coverage of biochemical functionality as compared to other pipelines. The RAST annotation pipeline is capable of associating only 2002 reactions and transporters to genes (compared to 2123 in the latest *E. coli* genome scale model [7]) and is therefore specialised at reproducing core metabolic and biosynthetic functionalities, particularly in line with its generalised biomass equations, but absent of less common biochemistry. In our case, this included most of the Wood-Werkman cycle and the ADP-dependent acetyl-CoA ligase as prime example of less common central carbon metabolism not predictable by this tool. It is therefore especially important to rely on experimental evidence to generate a well curated model from this pipeline.

1. Is the pipeline producing reproducible results

What is assumed but not necessarily true is that automated pipelines are behaving reproducibly. This should be tested before beginning reconstruction efforts, particularly if multiple models are to be compared, and annotated in a similar time frame so as to avoid any potential biases from updates. We found two subsequent annotations of the same organism and genbank file in the same day differed by 3 unique genes in each annotation and 11 differing gene start sites. The problem was eventually tracked down to a node which had failed to update, but shows the importance of running controls even in a computational environment. Similarly, a later annotation lost a substantial number of reactions with respect to an earlier annotation, which was the result of staged updates being processed to different elements of the pipeline at different times.

1. Gap filling curation

The first step to assess the automated annotation is to check the gap filling. Unlike the old ModelSEED pipeline, recent releases such as KBase allow for optional gap filling and the choice of a defined media. The gap filling algorithm should be considered as a recommendation and does not work on a guaranteed basis of adding the minimal number of reactions, nor is it reproducible as it can select equally viable alternate solutions. Even if no gene candidates can be found to allow for a function which must be present in the model, care should be taken to ensure the results are the most biologically reasonable. As an example, the weighted LP formulation used by KBase, which uses various metrics to weight preferred reaction candidates such as presence in KEGG, added an entirely new degradation pathway consisting of one transporter and three reactions to allow the the production of demethylmenaquinone from menaquinone 7 when performing a complete media based gap-fill for *P. acidipropionici* 4875. An alternative gap fill required the addition of three reactions to fix the biosynthetic pathway, one of these reactions being essential for the biosynthesis of isoprenoids; another gap. The differing use of cofactors often leads to alternative solutions without any preference.

In addition, gap-filling is limited to the accuracy of the biomass equation, commonly initially performed on the generic biomass templates supplied by the RAST pipeline. This template is influenced by the classification of the Gram type of the bacteria. In the case of *Propionibacterium*, the Gram type is incorrectly classified as negative which leads to the spurious addition of about 30 reactions to allow the production of lipid A. Recent updates to the RAST pipeline now allow the manual choice of template for the biomass equation, and this is recommended to avoid incorrect calling.

One final point is that the gap-filling algorithm can alter the reversibility of reactions. Because the free energy of reactions are inferred from a combination of component contribution type methods and heuristics in the RAST framework, errors in these predictions can lead to an incorrect assignment of reaction reversibility. In such circumstances, the ability of the gap-filling algorithm to alter the reaction directionality to reversible can be convenient, but it is important to ensure that degradation pathways have not been artificially turned into synthesis pathways or reactions have needlessly been reversed due to the minflux approximation. Note that this phenomenon wasn’t necessarily observed, but reaction directionalities in proline degradation had to be altered to ensure this pathway could not be used for biosynthesis. Gap-filled reactions were also added as reversible in KBase without consideration of prior annotation as irreversible at the time of writing; therefore the directionality of all newly added reactions should be reassessed.

1. Reactions may not be carried forward to model from annotated genes, and the depth of the RAST manual curation effort is insufficient to cover necessary biochemistry

Reactions belonging to subsystems may not be carried through into models in the presence of an associated FIGfam in the RAST framework. In these cases the genes theoretically encoding particular reactions have been annotated and the subsystem, as defined by the ModelSEED framework, has been marked as active. However, these reactions are not encoded in the subsequent model. These types of errors may in part be due to variant FIGfams which have not received the same linkage to the reaction, even if they are encoded in a subsystem; as for asparaginase. Another example is the citramalate synthase (2.3.1.182) which is present in the branched-chain amino acid biosynthesis subsystem and contains no FIGfam linkage. In at least the case of the cystathionine gamma synthase for methionine biosynthesis, this was because the reaction consuming o-phospho-L-homoserine to produce cystathionine was absent from the database. All pathways considered important for the purpose of a genome scale model must be manually inspected to ensure all reactions that should be in a subsystem are present and for the reasons discussed above, interactive tools such as the subsystems viewer from ModelSeed are not sufficient for this task.

An obvious but important consideration is that the depth of coverage of biochemistry by the RAST database is considerably small, linking approximately 1750 biochemical reactions to genes (excluding transporters). In the case of *Propionibacterium*, the propionate biosynthesis pathway lacked key enzymes including the carboxyltransferase (EC 2.1.3.1) and propionyl-CoA: succinyl-CoA CoA transferase (EC 2.8.3-). Another example is cysteine degradation which completely lacks any FIGfam associations and is therefore never encoded in RAST models without gap filling.

The most efficient way to catch these errors is to observe the list of genes annotated and their associated reactions and identify highly specific annotations (such as metabolic processes with full EC numbers) without a corresponding reaction assigned. The use of a pan-genomic matrix is appropriate for this purpose. Such a procedure will also assist with identifying biochemical functionalities not linked to any FIGfam. These results can be used to update the previous gap filling step.

1. Subsystems based pathway curation

The subsystem definitions in ModelSEED underpin the FIGfam annotation efforts and serve as a useful guide to assist the manual curation process. While the FIGfams are still involved in the KBase annotation pipeline, the subsystems are no longer immediately accessible through this framework. A subsystems based manual curation effort helps to identify genes that were missed or incorrectly called during the initial annotation, missing multifunctionalities (promiscuous activities) to attribute to enzymes and incorrect reaction directionalities. As an initial caution, genes annotated in the model can be marked as absent in the subsystem at a reasonably high frequency. For this reason, direct use of the activity of subsystems for bioinformatics-based comparisons is not useful, as previously discussed.

Stepping through the subsystems to identify uncalled or miscalled genes is a particularly useful exercise, especially from a pan-genomic approach where differences between strains can be further probed. Although slow, the BLAST tool within ModelSEED is particularly useful as it is not restricted to called genes as for NCBI BLAST searches and can be used to find genes missed by the gene calling algorithm or non-specific annotations. The use of local BLAST libraries and installations can greatly speed up the annotation procedure and eliminate the need to rely on the slow ModelSEED interface, especially when annotating multiple models at once. As demonstrated by the gene caller trials, the choice of gene caller can vary the start and stop points of a gene slightly which can make the difference between a specific annotation and a general annotation. Using the subsystems type approach can help identify these non-specific annotations.

In addition, the approach allows the curator to check whether all necessary reactions have been carried through into the model. While most FIGfams that lack the reaction association will have been dealt with in the previous step, the pipeline is particularly bad at annotating multifunctionalities to FIGfams. Examples include the promiscuous activities of enzymes associated with acetate metabolism that also act on propionate, such as the propionyl-CoA ligase. Other examples of missed multifunctionalities include the pyruvate:ferredoxin oxidoreductase which can also act on 2-oxobutyrate and propionyl-CoA, a common reaction required for the degradation of amino acids such as threonine to propionate. Another key example is the multifunctionality of reactions in the leucine biosynthetic pathway that allow the conversion of D-citramalate to 2-oxobutyrate. This forms one of two pathways to produce 2-oxobutyrate required for isoleucine biosynthesis. Because RAST is incapable of annotating this pathway all models produced must synthesise and degrade threonine to generate the 2-oxobutyrate precursor, an example where the depth of the subsystems effort has skewed all RAST-based annotations.

Finally, by stepping through subsystems, the reactions can be checked for correct directionality for subsystem functionality. Gap filling is capable of altering reversibility to allow growth and will typically correct irreversible biosynthetic reactions, except if alternative routes are present. This is possible for lysine synthesis where the 2,3,4,5-tetrahydropyridine-2,6-dicarboxylate N-succinyltransferase is irreversible in the wrong direction. More focus needs to be placed on the degradation pathways as these are not likely to be corrected by the gap filling process. Additionally, these pathways should be checked to ensure the lower energy degradation pathways are not completely irreversible and can be used for biosynthesis, as described previously. For example, the ornithine carbamoyltransferase reaction is irreversible in the wrong direction and therefore prevents the arginine deiminase pathway from functioning, while proline degradation includes spontaneous reaction steps and should not be reversible like the default annotation.

It is recommended that a pathway by pathway approach is taken for curating the model using both the SEED subsystems and the MetaCyc [8] pathways which contain detailed descriptions, links to UniProt to assist in BLAST searching and a greater amount of biochemistry. In addition, the reactions in SEED should be capable of attaining the physiological directions as annotated in MetaCyc. Both MetaCyc and BRENDA [9] are useful resources for identifying multifunctional enzymatic activities.

1. Identification of incorrect reactions, GPRs and cofactor balancing.

Incorrect associations between reactions and FIGfams are more difficult to detect, but at least one error has been identified based on an inconsistent functionality associated with metabolism. A proton pumping hydrogenase (rxn10118) is incorrectly associated with just the Cytochrome d ubiquinol oxidase subunit I FIGfam. Additionally, GPR annotations should be reviewed particularly for known enzymatic complexes for consistency. For example, the 2-oxoglutarate synthase reaction (rxn05939) contains a small and large subunit which are annotated with an “or” relationship instead of an “and” relationship, incorrectly implying the presence of one of these subunits allows catalysis of the reaction. The reconstruction contains hundreds of examples of poor GPR definitions.

In addition, RAST may not have the capability to identify the full impact of pseudogenes resulting from frameshifts in the annotation. This appears to be the result of a decision logic where if the majority of a complex is identified, then the functionality is added despite critical missing components. This was the case for cytochrome C oxidase which had full functionality in the genomic reconstruction of *P. acidipropionici* 4875 despite the absence of the cytochrome C oxidase polypeptide I, which is absent due to a documented frameshift mutation in a conserved functional domain [10]. Similarly, the ability to reduce nitrate separates the *P.* *freudenreichii* subspecies from *P.* *shermanii*, yet the RAST and KBase pipelines still correct and overcome this frameshift despite a reported frameshift in a subunit of the nitrate reductase. While potentially useful in specific circumstances, the apparent complex gap filling function of RAST can clearly result in the over-prediction of functionality and is a feature to be aware of.

Traditionally the RAST database appears to have been populated with a number of unbalanced reactions. These typically appear to result from population of the database with reactions from other databases where there is no ability to match the metabolites, for example, polymeric type compounds of undefined length. In such circumstances the undefined chemicals are simply absent from the equation. It is unclear whether these reactions may be utilised when gap filling, but the KBase reaction database appears to have been pruned of these reactions. At least one reaction that is unbalanced has been found to be associated with a FIGfam and remains in the KBase database, rxn04386, which phosphorylates threonine without a phosphate donor. This reaction is present in 880 models at the time the analysis was performed but was not found in the *Propionibacterium* annotation. We additionally found proton imbalances in several peptide transporters.

Cofactors also require specialised attention. The use of cofactors may be variable between species for the same reaction or be exclusively assigned to a single cofactor which may not be coupled to the metabolic model. For example, we re-annotated pyruvate oxidase as menaquinone-dependent rather than ubiquinone-dependent based on its expression in anaerobic fermentation conditions. Cofactors can be generally assigned, particularly for the lactate dehydrogenase where many cofactors are assigned to the same gene and these need to be manually assessed on a case by case basis. The ability of cofactors to be balanced should also be assessed. For example, an azurin-dependent nitrite reductase is associated with a subsystem in the RAST framework, but there are no reactions in the database that allow regeneration of this cofactor. We also added a ferredoxin-NADP oxidoreductase to allow regeneration of this cofactor in our model.

A final potential source of error is the incorrect annotation of complexes with a high degree of homology. For example, we found wide-spread misannotation of the 2-oxoacid dehydrogenase complexes, including the pyruvate dehydrogenase and branched-chain amino acid dehydrogenase.

# S12: Transcriptomics visualisation for relevant genes

Transcriptional data for relevant genes is displayed below using GBrowse [11]{Formatting Citation}. Data is collected from a complex fermentation of *P. acidipropionici* 4875 in exponential phase on 3 different carbon sources; glucose, sucrose and glycerol, performed in biological duplicates.


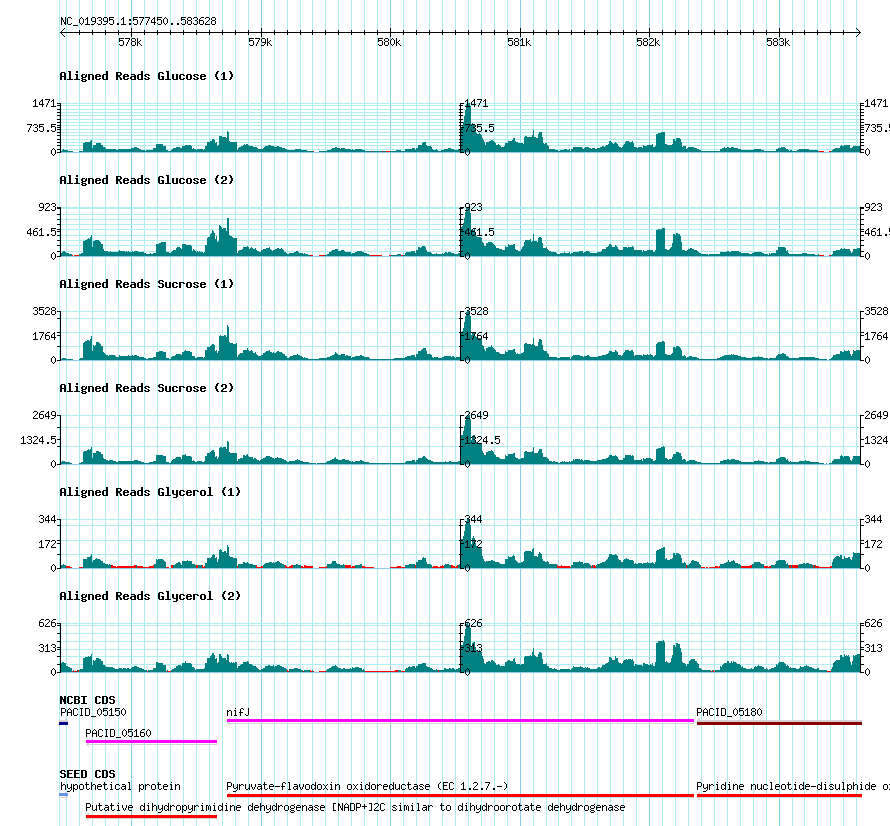


Figure 1: Transcription of the pyruvate-ferredoxin oxidoreductase (PFOR) with three different carbon sources, repeated in duplicate. The ferredoxin/flavodoxin produced by this enzyme must be regenerated and is apparently disconnected from the rest of metabolism, despite being associated with the production of a key fermentation product, acetate. We speculate the flanking genes, annotated as dihydroorotate dehydrogenase (PACID_05160) and pyridine nucleotide-disulfide oxidoreductase associated with PFOR (PACID_05180) in the annotation on NCBI may form a complex with electron bifurcating properties. Similarity of the dihydroorotate dehydrogenase to the type II variant, which uses quinones as a cofactor, leads us to speculate this complex may allow for the energetically unfavourable reduction of NAD with a quinol. The co-transcription of all of these genes lends support to our speculation.

# References

1. Contreras-Moreira, B.; Vinuesa, P. GET_HOMOLOGUES, a Versatile Software Package for Scalable and Robust Microbial Pangenome Analysis. *Appl. Environ. Microbiol.* **2013**, *79*, 7696–7701, doi:10.1128/AEM.02411-13.

2. Overbeek, R.; Olson, R.; Pusch, G.D.; Olsen, G.J.; Davis, J.J.; Disz, T.; Edwards, R.A.; Gerdes, S.; Parrello, B.; Shukla, M.; et al. The SEED and the Rapid Annotation of microbial genomes using Subsystems Technology (RAST). *Nucleic Acids Res.* **2014**, *42*, 206–214, doi:10.1093/nar/gkt1226.

3. Brettin, T.; Davis, J.J.; Disz, T.; Edwards, R.A.; Gerdes, S.; Olsen, G.J.; Olson, R.; Overbeek, R.; Parrello, B.; Pusch, G.D.; et al. RASTtk: A modular and extensible implementation of the RAST algorithm for building custom annotation pipelines and annotating batches of genomes. *Sci. Rep.* **2015**, *5*, 8365, doi:10.1038/srep08365.

4. Aziz, R.K.; Bartels, D.; Best, A.A.; DeJongh, M.; Disz, T.; Edwards, R.A.; Formsma, K.; Gerdes, S.; Glass, E.M.; Kubal, M.; et al. The RAST Server: rapid annotations using subsystems technology. *BMC Genomics* **2008**, *9*, 75, doi:10.1186/1471-2164-9-75.

5. Zerbino, D.R.; Birney, E. Velvet: Algorithms for de novo short read assembly using de Bruijn graphs. *Genome Res.* **2008**, *18*, 821–829, doi:10.1101/gr.074492.107.

6. Bankevich, A.; Nurk, S.; Antipov, D.; Gurevich, A. a.; Dvorkin, M.; Kulikov, A.S.; Lesin, V.M.; Nikolenko, S.I.; Pham, S.; Prjibelski, A.D.; et al. SPAdes: A New Genome Assembly Algorithm and Its Applications to Single-Cell Sequencing. *J. Comput. Biol.* **2012**, *19*, 455–477, doi:10.1089/cmb.2012.0021.

7. Orth, J.D.; Conrad, T.M.; Na, J.; Lerman, J. a; Nam, H.; Feist, A.M.; Palsson, B.Ø. A comprehensive genome-scale reconstruction of Escherichia coli metabolism--2011. *Mol. Syst. Biol.* **2011**, *7*, 535, doi:10.1038/msb.2011.65.

8. Caspi, R.; Altman, T.; Billington, R.; Dreher, K.; Foerster, H.; Fulcher, C.A.; Holland, T.A.; Keseler, I.M.; Kothari, A.; Kubo, A.; et al. The MetaCyc database of metabolic pathways and enzymes and the BioCyc collection of Pathway/Genome Databases. *Nucleic Acids Res.* **2014**, *42*, D459-71, doi:10.1093/nar/gkt1103.

9. Scheer, M.; Grote, A.; Chang, A.; Schomburg, I.; Munaretto, C.; Rother, M.; Söhngen, C.; Stelzer, M.; Thiele, J.; Schomburg, D. BRENDA, the enzyme information system in 2011. *Nucleic Acids Res.* **2011**, *39*, D670-6, doi:10.1093/nar/gkq1089.

10. Parizzi, L.P.; Grassi, M.C.B.; Llerena, L. a; Carazzolle, M.F.; Queiroz, V.L.; Lunardi, I.; Zeidler, A.F.; Teixeira, P.J.P.L.; Mieczkowski, P.; Rincones, J.; et al. The genome sequence of Propionibacterium acidipropionici provides insights into its biotechnological and industrial potential. *BMC Genomics* **2012**, *13*, 562, doi:10.1186/1471-2164-13-562.

11. Stein, L.D.; Mungall, C.; Shu, S.; Caudy, M.; Mangone, M.; Day, A.; Nickerson, E.; Stajich, J.E.; Harris, T.W.; Arva, A.; et al. The Generic Genome Browser : A Building Block for a Model Organism System Database. **2002**, 1599–1610, doi:10.1101/gr.403602.12.
